# Enhancing Antioxidant and Antimicrobial Activities in Bee-Collected Pollen through Solid-State Fermentation: A Comparative Analysis of Bioactive Compounds

**DOI:** 10.3390/antiox13030292

**Published:** 2024-02-27

**Authors:** Adriana Cristina Urcan, Adriana Dalila Criste, Daniel Severus Dezmirean, Otilia Bobiș, Victorița Bonta, Ramona Flavia Burtescu, Neli-Kinga Olah, Mihaiela Cornea-Cipcigan, Rodica Mărgăoan

**Affiliations:** 1 Department of Microbiology and Immunology, Faculty of Animal Science and Biotechnologies, University of Agricultural Sciences and Veterinary Medicine, 400372 Cluj-Napoca, Romania; adriana.criste@usamvcluj.ro; 2 Department of Beekeeping and Sericulture, Faculty of Animal Science and Biotechnologies, University of Agricultural Sciences and Veterinary Medicine, 400372 Cluj-Napoca, Romania; ddezmirean@usamvcluj.ro (D.S.D.); obobis@usamvcluj.ro (O.B.); victorita.bonta@usamvcluj.ro (V.B.); 3 PlantExtrakt Ltd., Rădaia, 407059 Cluj-Napoca, Romania; ramona.burtescu@plantextrakt.ro (R.F.B.); neli.olah@plantextrakt.ro (N.-K.O.); 4 Faculty of Pharmacy, “Vasile Goldiş” Western University of Arad, 310414 Arad, Romania; 5 Department of Horticulture, Faculty of Horticulture and Business in Rural Development, University of Agricultural Sciences and Veterinary Medicine, 400372 Cluj-Napoca, Romania; 6 Department of Animal Production and Food Safety, Faculty of Veterinary Medicine, University of Agricultural Sciences and Veterinary Medicine, 400372 Cluj-Napoca, Romania; rodica.margaoan@usamvcluj.ro

**Keywords:** bee-collected pollen, fermentation, lactic acid bacteria, polyphenols, amino acids, antioxidant activity, antimicrobial activity

## Abstract

The present study investigates the impact of solid-state fermentation on bee-collected pollen using a consortium of *Lactobacillus plantarum*, *Apilactobacillus kunkeei*, and *Lactobacillus acidophilus*. Another aim is to compare the nutritional and bioactive properties of natural versus fermented pollen, focusing on macronutrient composition, pH, acidity, lactic acid content, and profiles of polyphenolics and flavonoids. Our results indicated significant enhancements in the contents of amino acids, suggesting improved protein content, alongside increases in polyphenolic and flavonoid contents post-fermentation. According to the heat mapping and cluster analysis, increased antioxidant and antimicrobial activities against Gram-positive and Gram-negative bacteria, particularly *E. coli*, were observed in the fermented bee-collected pollen samples, which may have been due to the accumulation of phenolic compounds (e.g., ellagic acid, kaempferol, quercetin, and quercetin-3-*O*-rutinoside). Furthermore, significant positive correlations of the fermented bee-collected pollen samples with non-essential amino acids were recorded compared with the unfermented bee-collected pollen samples, which may have been due to the fermentation process and the conversion of proteins into free amino acids via proteolysis. Future research could explore the underlying mechanisms, the scalability of fermentation, its application in functional foods, and the health benefits of fermented bee-collected pollen in human diets.

## 1. Introduction

At present, there is a general consensus that maintaining good health relies heavily on a balanced diet and lifestyle. Over time, consumer attitudes toward food production and consumption have evolved, leading to the integration of functional foods as a significant component of the food market [1]. Modern consumers are becoming more concerned regarding the excessive usage of synthetic pharmaceuticals and tend to be oriented toward natural products that include nutrients and biologically active compounds from natural sources, such as plants or bee products. These products provide a variety of health advantages, such as antibacterial, antifungal, antiviral, anti-inflammatory, antioxidant, anticancer, and hepatoprotective activities [2,3,4].

Bee-collected pollen (BP), a complex mixture of plant pollen, nectar, and honeybee enzymes, is recognized for its substantial nutritional and therapeutic properties. Rich in carbohydrates, proteins, amino acids, lipids, vitamins, and other bioactive compounds such as flavonoids and polyphenols, BP is valued as a powerful dietary supplement that offers a multitude of health benefits, including antioxidant, anti-inflammatory, and immune-boosting properties [5,6,7]. Its complex nutritional profile recommends it as a functional food; however, the bioavailability of these nutrients and bioactive compounds can be limited due to its resilient microstructure, predominantly the exine layer, which is designed to safeguard plant genetic material [8,9,10]. For this reason, researchers have looked for ways to release nutrients and bioactive substances from BP grains, inspired by the natural fermentation process from the beehive, through which BP turns into bee bread, eventually leading to lactic acid fermentation [11,12,13]. Consequently, fermenting BP is advocated as a strategy to augment its quality as a functional product, offering additional value over unfermented BP [14]. In recent years, researchers have been experimenting with initiating BP fermentation at a laboratory scale by introducing various microorganisms, such as *Lactobacillus plantarum*, *Lactobacillus rhamnosus*, *Lactobacillus acidophilus*, *Lactobacillus casei*, or *Lactobacillus paracasei* under different conditions [15,16,17,18,19]. Fermentation, particularly with probiotic strains like *Lactobacillus plantarum*, has been proposed by other authors as a strategy to enhance the nutritional value and bioavailability of BP compounds [20]. *Lactobacillus plantarum*, with its robust fermentative ability and adaptability to different substrates, has been documented to improve the digestibility and nutritional profile of various foods. The fermentation process with *L. plantarum* and *L. acidophilus* not only breaks down complex food matrices but also potentially increases the levels of essential amino acids, vitamins, and bioactive peptides, adding to the functional value of BP [21,22,23,24,25]. One of the notable benefits of *L. plantarum* in fermentation is its capacity to improve the bioavailability of nutrients. This attribute is particularly valuable in the context of fermenting nutrient-dense but biologically inaccessible foods like BP. Additionally, *L. plantarum* is known for its probiotic potential, which contributes to the health benefits of fermented products [26].

*L. plantarum* fermentation boosts the antioxidant properties of fermented bee-collected pollen (FBP), increasing its total phenolic content and enhancing its antioxidant capacity. This suggests the potential for preventive nutrition. *L. plantarum*’s probiotic properties, combined with BP’s nutrients, offer a potent health-promoting product, improving gut health and immune system modulation [13,27]. The efficiency of fermentation has been proven by several authors. Kaškonienė et al. [28] demonstrated that *Lactobacillus rhamnosus* GG fermentation significantly increased the flavonoid content by 55–135%, enhancing the total phenolic content and antioxidant activity in BP. Another study carried out by Hernández-Alcántara et al. [22] found that solid-state fermentation with *L. rhamnosus* improved the phenolic content, flavonoid content, and antioxidant bioavailability in BP. Shirsat et al. [29] showed that *L. lactis* fermentation boosted BP’s nutritional value. Spontaneous and inoculated BP fermentation increased polyphenol contents and antioxidant activity [16]. Poyraz et al. [30] used *Lactobacillus kunkeei* and yeasts for fermentation, enhancing BP’s characteristics and digestibility. *Lactobacillus plantarum* and *Saccharomyces cerevisiae* fermentation increased BP’s nutrient availability, total phenolics, and antioxidant activity. Scanning electron microscopy (SEM) and differential scanning calorimetry (DSC) analyses suggested structural modifications, potentially increasing digestive absorption, thus enriching BP’s nutritional and probiotic profile [31]. The current state of knowledge underscores the potential of lactic acid bacteria (LAB) in transforming BP into a more nutritive and bioavailable form, thereby enhancing its functional properties [32,33,34,35,36]. While most of the existing studies have explored the fermentation of BP with individual strains, the present study aimed to use a consortium of *Lactobacillus plantarum*, *Apilactoobacillus kunkeei*, and *Lactobacillus acidophilus* for the fermentation. The purpose of this study was to elucidate the impact of fermentation with *Lactobacillus plantarum, Apilactobacillus kunkeei*, and *Lactobacillus acidophillus* on the nutritional and bioactive properties of BP. This study aims to provide a detailed comparative analysis of the macronutrient composition, pH, acidity, lactic acid content, polyphenolic and flavonoid profiles, and the antioxidant and antimicrobial activities of BP before and after the fermentation process. 

## 2. Materials and Methods

### 2.1. Chemicals and Reagents

All chemicals employed in the experiments were of analytical-grade purity and were procured from Sigma-Aldrich and Merck (Merck KGaA, Darmstadt, Germany, and/or its affiliates). For the preparation of standards and mobile phases, Milli-Q water with a pH of 2.4 (adjusted with o-phosphoric acid) was utilized. Additionally, all reagents and sample extracts underwent filtration through a 0.45 μM MF-Millipore™ Membrane Filter from Merck (Darmstadt, Germany).

### 2.2. Samples

Five (BP) samples were obtained from apiaries located in Cluj-Napoca County, northwest Transylvania, Romania during the flowering season in May–September 2022. The BP samples were collected using pollen traps, cleaned of any pieces of wood or bees, and stored in the freezer at −18 °C until they were analyzed. Before performing the analyses, the samples were homogenized and powdered using a lab-type blender. 

### 2.3. Samples Preparation

The BP samples, before and after fermentation, each containing 5 g, along with 25 mL of 70% ethanol, were subjected to individual extraction using an ultrasonic bath operating at 30 °C for 60 min. This ultrasonic bath was a Bandelin Sonorex, specifically the Sonorex Super RK 100 H model, manufactured by Bandelin Electronic GmbH and Co, KG, Berlin, Germany. Following the sonication process, the resultant mixture underwent centrifugation at 15,269 times the force of gravity (15,269 g) for 10 min. Subsequently, the supernatants were carefully separated and preserved at a temperature of −4 °C until further analysis.

### 2.4. Microscopic Analysis

The analysis of the botanical origin of the BP samples was conducted according to the method described by Louveaux et al. [37], without acetolysis and adapted for BP [38]. To identify the pollen type in the BP samples, either a pollen atlas or reference slides prepared from flower anthers were used [39]. Taxonomic identification was performed at the most specific level possible, taking into account any encountered difficulties. The palynological analysis was carried out using an Olympus BX51 optical microscope at a magnification of 400×.

### 2.5. Induction of Fermentation and Monitoring of Lactic Acid Bacteria’s Growth

#### 2.5.1. Lactic Acid Bacteria (LAB) 

Three LAB strains were used for in this study: *Lactobacillus plantarum* ATCC 8014, *Apilactobacillus kunkeei* ATCC 700308, and *Lactobacillus acidophilus* ATTC 700396. These cultures were selected because it has already been demonstrated in previous studies [16,31,35,40,41,42,43] that each strain, individually, presented good results in terms of BP fermentation. Both *L. acidophilus* and *L. plantarum* are well-documented probiotics appropriated for the solid-state fermentation of BP [15,17,31]. Incorporating *A. kunkeei*, a strain that is naturally found in bee products and is well-adapted to the high sugar content of BP, will lead to a more stable and efficient fermentation process. Studies have shown [44] that microbes that are naturally associated with a substrate often lead to more effective and targeted fermentation processes. Each LAB strain was statically grown in MRS broth with Tween 80 (Biolife, Italy) under anaerobic conditions at 37 °C or 30 °C until the cell density, measured spectrophotometrically at a wavelength of 600 nm, reached 0.730, corresponding to 2.92 × 10^9^ CFU/mL for each strain. The final inoculum used for the fermentation of BP was a mix of *L. plantarum*, *A. kunkei*, and *L*. *acidophillus*, maintaining a ratio of 1:1:1, with each strain contributing an equal cell density of 2.92 × 10^9^ CFU/mL. This balanced approach ensured a synergistic interaction among the bacterial strains, promoting an effective fermentation process. To prevent decreased biomass production, prior to fermentation, 1 mL of the LAB mix was suspended in a solution containing 1 g of BP and 9 mL of MRS broth [31]. The mixture was then incubated at 35 °C for 24 h under anaerobic conditions. 

#### 2.5.2. Fermentation of BP

Furthermore, the BP substrate was prepared under aseptic conditions after it had been previously pasteurized at 85 °C for 20 min. First, 100 g of each BP sample was prepared by employing a 2:1 ratio of BP and water. The substrates were placed in 200 mL flasks, which had previously been sealed and sterilized at 121 °C for 15 min. Subsequently, the flasks were inoculated with the LAB mixture and incubated at 3 5 °C for 72 h. This temperature was chosen based on the optimal growth rates of the LAB strains involved, ensuring their metabolic activity and stability throughout the fermentation period. To optimize the fermentation environment, the substrate-to-inoculum ratio was maintained at 10:1 (*w*/*v*). This proportion was determined to be optimal for facilitating nutrient availability and microbial activity. Each sample was prepared in three replicates. 

#### 2.5.3. pH and Acidity

Potentiometry was used to determine the pH and titratable acidity, in accordance with [41] with slight changes. First, 5 g of each sample was introduced to 30 mL of ultrapure water and stirred for 2 min at 400 rpm. An automated titrator (TitroLine 5000 with A7780 electrode, Roth, Germany) and a 0.5 N NaOH standard solution were used for the titration process. The findings were presented as equivalents of NaOH/kg^−1^. The percentage of lactic acid was determined by transforming the acidity [29] according to Formula (1):

(1) $$Lactic\;acid\;content=\left(\frac{V_{NaOH}\times C_{NaOH}\times 0.09}{m}\right)\times 100$$

where *V_NaOH_* is the the volume of NaOH used for the titration, *C_NaOH_* is the concentration of NaOH, *m* is the mass of the sample, and 0.09 is the equivalent weight of lactic acid.

#### 2.5.4. Lactic Bacteria Count

Lactic acid bacteria were identified by counting on a plate, using De Man Rogosa and Sharpe agar (MRS) as the medium (Sigma-Aldrich, Germany). Eight serial decimal dilutions were performed. Briefly, 10 mL of peptone salt solution (8.5 g/L) was used to suspend 1 g of each BP sample, which was then vortexed for 10 s. Following this, 0.1 mL aliquots of each dilution were put on Petri dishes, which were then covered with sterile MRS medium. Using a polycarbonate anaerobic jar (Oxoid, Basingstoke, UK) and disposable CO_2_-producing envelopes for anaerobiosis (Thermo Fisher Scientific, Waltham, MA, USA), the plates were incubated under anaerobiosis for 72 h at 37 °C. After the incubation period, colonies were identified and counted, and the results were expressed as CFU/g. Furthermore, the dynamic of the mix of lactic acid bacteria during fermentation was measured spectrophotometrically at a 600 nm wavelength. Finally, 0.1 mL aliquots of each dilution were put in liquid MRS medium and incubated at 37 °C for 48 h.

### 2.6. Nutritional Analysis

#### 2.6.1. Moisture Content

Initially, 1 g of each sample was weighed and heated at 103 ± 2 °C for 2 h. After cooling, it was weighed again and then heated repeatedly until a constant weight was achieved [45]. 

#### 2.6.2. Ash Content

Ash determination was conducted through gravimetric analysis following incineration in an oven at 600 °C until a constant weight was attained [46].

#### 2.6.3. Total Nitrogen Content

The Kjeldahl technique was used to determine the total nitrogen concentration. Boric acid was utilized to quantify the nitrogen content from the samples. For quantification, the N × 6.25 conversion factor [43] was applied.

#### 2.6.4. Sugars Determination

Bonta et al.’s [47] HPLC-IR method was modified for BP samples and utilized for the determination of free sugar concentration. A Shimadzu Liquid Chromatograph type SLC-10 Avp equipped with an HPLC/IR refractive index detector was used along with an Altima Amino 100 stainless steel column (Alltech, Nicholasville, KY, USA) for the separation of sugars by chromatography. Acetonitrile and water (75:25 *v*/*v*) were used as the mobile phase, with a flow rate of 1.3 mL/min. There was a 10 µL injection volume. The calculation was based on calibration curves for standard solutions with various concentrations (0.5–80 mg/mL) for each sugar. By comparing the resulting peak area with those of reference sugars, the sugars were quantified. The results were given in g/100 g BP.

Quantification of total carbohydrates and energetic value: The following method was used to estimate the total amount of carbohydrates: Total carbohydrates = 100 − (g ashes + g proteins + g lipids). The energy value was determined in accordance with [48] utilizing formula 2 for calculation.

Energy (kcal) = 4.1 × (g protein + g carbohydrates) + 9.3 × (g fat)(2)

### 2.7. Free Amino Acid Analysis

First, 0.25 g of the sample was dissolved in 10 mL of ultrapure water; the sample was sonicated for 30 min at 40 KHz using an Ultrasonic Cleaner from Sonica (Milan, Italy) and then centrifuged for 10 min at 10,000 rpm. Following the methods outlined in the amino acids EZ:Faast kit procedure (Phenomenex, Torrance, CA, USA) comprising 3 steps—solid-phase extraction, derivatization, and liquid–liquid extraction—25 μL of sample was extracted from the supernatant. Using a Shimadzu 2010 EV (Kyoto, Japan) equipped with a quaternary pump, column oven degasser, automatic injector with cooling system and autosampler, LC-MS nitrogen generator, and two detectors—one with an SPD-M-20A photodiode network and the other an LC-MS 2010 EV mass spectrometer with an electrospray interface—the profile of the free amino acids was ascertained by LC-MS. The method’s operational parameters were as follows: 10 mM ammonium in water (A) and 10 mM ammonium in methanol (B) for the mobile phase; stationary phase: an EZ chromatographic column with dimensions of 250 × 3.0 mm. The parameters were as follows: injection volume: 1 µL; detector voltage: 1.7 KV; acquisition time: 33 min; flow rate: 0.3 mL/min; column temperature: 35 °C. The internal standard approach was used for the identification and quantification of amino acids. The values of each free amino acid concentration were represented by the results, which were stated as mg/100 g sample. The operational parameters of the method were as follows: stationary phase: EZ chromatographic column AAA-MS, 250 × 3.0 mm, mobile phase: 10 mM ammonium in water (A) and 10 mM ammonium in methanol (B). The flow rate was 0.3 mL/min, the column temperature was 35 °C, the injection volume was 1 µL, the detector voltage was 1.7 KV, and the acquisition time was 33 min. The identification and quantification of amino acids was carried out using the internal standard method. The results were expressed as mg/100 g sample, representing the values of each free amino acid concentration.

### 2.8. Total Phenolic Content (TPC)

The Folin–Ciocâlteu technique [49] was used to calculate the total phenolic content. First, 100 µL of Folin–Ciocâlteu reagent (previously diluted 1:10 with deionized water; 0.2 M with respect to acid) was mixed with 10 µL of each BP and FBP extract. Following this, 80 µL of a 1 M sodium carbonate (Na_2_CO_3_) solution was added. The combination was then allowed to react for 15 min, at which point the absorbance at 765 nm was measured. Based on a calibration curve of a gallic acid solution with concentrations ranging from 0.025 to 0.15 mg/mL (R^2^ = 0.998), quantification was carried out. The experiments were conducted in triplicate, using a 96-well microplate reader (Synergy™ HT BioTek Instruments, Winooski, VT, USA).

### 2.9. Total Flavonoid Content (TFC) 

The determination of total flavonoid content was conducted spectrophotometrically following the protocol outlined by Mărghitaș et al. [3]. In this method, each tested sample (25 µL) was diluted with 100 µL of ultrapure water. Subsequently, 10 µL of 5% NaNO_2_ solution was added, followed by the addition of 15 µL of 2% AlCl_3_ solution after 5 min of incubation. The mixture was further treated with 50 µL of 1 M NaOH and an additional 50 µL of ultrapure water after an additional 6 min. The absorbance of the resulting solution was measured at 510 nm. Quantification of flavonoids was performed using a calibration curve generated from a series of quercetin solutions with concentrations ranging from 0.025 to 0.2 mg/mL (R^2^ = 0.999). The results were expressed as milligrams of quercetin equivalents (Qe) per gram of dry matter sample. All experiments were conducted in triplicate using a 96-well microplate reader (Synergy™ HT BioTek Instruments, Winooski, VT, USA).

### 2.10. Individual Polyphenolic Compounds

A Shimadzu Nexera I LC/MS-8045 (Kyoto, Japan) UHPLC system, equipped with an ESI probe and quadrupole rod mass spectrometer, as well as a quaternary pump and an autosampler, was utilized to analyze the BP extracts. Formic acid was utilized as an organic modifier in the mobile phase, which was a gradient of ultrapure water and methanol (Merck, Darmstadt, Germany). The initial gradient composition was 5:90:5 methanol, water, and formic acid water, respectively. The methanol and formic acid were both of LC/MS purity. The analysis of phenolic chemicals in the BP and FBP extracts was conducted using an injection volume of 100 µL and a flow rate maintained at 0.5 mL/minute. A Luna C18 reverse-phase column (150 mm × 4.6 mm × 3 mm, 100 Å) from Phenomenex (Torrance, CA, USA) was used for the separation, and its temperature was adjusted to 40 °C. A quadrupole rod mass spectrometer equipped with electrospray ionization (ESI) in both positive and negative MRM (multiple reaction monitoring) ion modes was used for the detection process. A 300 °C interface temperature was used, and 10 L/min of nitrogen gas was used for drying and vaporization at 35 psi. By comparing the MS spectra and transitions between isolated chemicals and standards, substances were identified. For each material, the primary transition from its MS spectra was utilized for identification and quantification. Calibration curves were used for the quantification process. The assay was carried out three times, and the results were expressed as mg/g of dry matter sample.

### 2.11. Antioxidant Activity

#### 2.11.1. Determination of DPPH Scavenging Activity (DPPH Method)

Using a spectrophotometric approach, the scavenging activity of the BP and FPB samples against the 2,2-diphenyl-1-picrylhydrazyl radical (DPPH) was assessed [49]. Briefly, 200 µL of DPPH solution (0.02 mg/mL) was mixed with 40 µL of appropriately diluted extracts. The samples’ absorbance was measured at 517 nm after 15 min. For measurement, a Trolox calibration curve (R^2^ = 0.997) was created using solutions ranging from 0.01 to 0.1 mM. The activity of radical scavenging is measured in milligrams of Trolox equivalents per gram of sample (mmol Trolox equivalent/g dry matter sample).

#### 2.11.2. Determination of Trolox Equivalent Antioxidant Capacity (TEAC Method)

The Trolox equivalent antioxidant assay was carried out, with minor changes, in accordance with the method reported by Margaoan et al. [38]. This is based on the scavenging of the 2,2′-azino-bis (3-ethylbenzothiazoline-6-sulfonic acid) radical (ABTS^•+^), turning it into a colorless product. The ABTS^•+^ cation radical was formed as a result of the reaction between a 2.45 mM potassium persulfate solution and a 7 mM ABTS solution. Before measuring, the ABTS^•+^ solution was diluted with ethanol, resulting in an absorbance of 0.700 ± 0.025 at 734 nm. For the test, 30 µL of each sample and 170 µL of the resulting solution were mixed. The absorbance was tested six minutes later. The standard calibration curve was linear between 0.04 and 0.4 mg of Trolox (R^2^ = 0.998). The results were expressed in milligrams of Trolox equivalents per gram of sample (mg Trolox equivalent/g dry matter sample).

#### 2.11.3. Determination of Ferric-Reducing/Antioxidant Power (FRAP Method)

The approach outlined by Cornea-Cipcigan et al. [50] was utilized to calculate ferric-reducing/antioxidant power (FRAP), with modifications required by the matrix under investigation. First, 300 µL of FRAP reagent, 10 µL of ultrapure water, and 10 µL of each BP extract were combined. For five minutes, the samples were incubated at 37 °C. Using a standard calibration curve (R^2^ = 0.998) and known quantities of aqueous Fe^II^ solutions (0.1–1 mmol/L of FeSO_4_·7H_2_O), the antioxidant capacity was determined by comparing the reaction signals. The results were reported as mmol/g Fe^II^ dry matter sample after the absorbance was measured at 593 nm.

### 2.12. Antimicrobial Activity 

The antimicrobial activity of the BP and FBP samples was evaluated by the disk diffusion method and microdilution method [51,52]. The selected bacterial strains were as follows: *Staphylococcus aureus* (ATCC 25923), *Enterococcus faecalis* (ATCC 29212), *Escherichia coli* (ATCC 25922), *Pseudomonas aeruginosa* (ATCC 27853), and *Candida albicans* (ATCC 10231).

#### 2.12.1. Disk Diffusion Method

First, 0.5 mL of each microbial suspension was inoculated on Petri dishes with MH agar plates, and SDA agar for *Candida* species, after the suspensions were adjusted to a concentration of 0.5 McFarland. After removing any leftover liquid, the agar surface was left to dry for 15 to 20 min at 35 °C. Following that, aseptic wells were formed, and 20 µL of each sample was added to each well. For Gram-positive bacteria, a positive control amoxicillin disk (30 μg/mL) was employed, while for Gram-negative bacteria, a norfloxacin disk (10 μg/mL) was used. Additionally, for yeast, a miconazole disk (10 μg/mL) served as the positive control. Subsequently, the plates were incubated at 37 °C for 24 h for bacteria, and at 28 °C for 48 h for the fungal strain. Following incubation, the diameters of the inhibition zones (in mm) were measured. The analysis was conducted in triplicate.

#### 2.12.2. Determination of the Minimum Inhibitory Concentrations (MICs) 

The MICs were determined using a serial microdilution technique in Mueller–Hinton broth supplemented according to species, with a final microorganism suspension of 0.5 McFarland. Amoxicillin served as the positive control for Gram-positive bacteria, norfloxacin for Gram-negative bacteria, and miconazole for yeast. Additionally, untreated bacteria were included as negative controls. Following 24 h of incubation at 37 °C, the plate was examined at 600 nm using a BioTek Synergy 2 multichannel spectrophotometer (BioTek Instruments, Winooski, VT, USA). The MIC for each microorganism was defined as the lowest concentration that exhibited 100% inhibition of microbial growth. The analysis was performed in triplicate.

### 2.13. Statistical Analysis

All determinations were conducted in three independent replicates, and the results obtained were expressed as the mean ± standard deviation (SD). Data analysis was performed using one-way analysis of variance (ANOVA), followed by Tukey’s multiple range test, using GraphPad Prism version 10 (San Diego, CA, USA); differences were considered significant at *p* < 0.05. Correlograms of Pearson’s correlation coefficients were constructed to evaluate the associations between the nutritional values, along with essential and non-essential amino acids of the BP and fermented BP samples, using the *corrplot* package. Heatmaps and dendrograms were generated using the Euclidean distance, with complete linkage to emphasize the similarities and differences in the fermentation process, biologically active substances, and bioactivities of the unfermented and fermented BP samples using the *Cluster R*, *ggplot*, *dendextend,* and *complexheatmap* packages from R (version 4.0.5). 

## 3. Results

### 3.1. Botanical Origin of BP Samples

Palynological analysis of BP reveals information about the botanical origin of the BP. Table 1 illustrates the families, genera, and species to which the analyzed BP samples belonged. 

According to the palynological analysis, most of the BP samples proved to be multifloral, with the exception of BP2 (Brassicaceae, *Brassica* sp.) and BP3 (Salicaceae, *Salix* sp.), which proved to be monofloral, with the dominance of a specific family exceeding 45%. The diversity in pollen types proved to be significant, with the highest diversity recorded in BP1, with 15 pollen types, and the lowest diversity in BP4, with 4 pollen types. The divergence in pollen types may vary according to the region of collection and available vegetation at the time of collection.

### 3.2. BP Fermentation—Kinetics of Bacterial Growth, pH, Acidity, and Lactic Acid Production

In order to achieve a successful fermentation process and ensure the growth of beneficial microorganisms that result in a high-quality fermented product, it is imperative to monitor pH and acidity during BP fermentation. The pH and acidity of the BP and FBP samples are visualized in Table 2.

**Figure 1:**
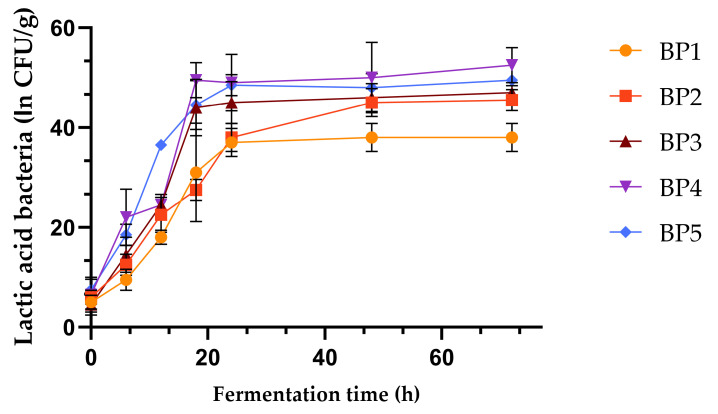
Kinetic monitoring of lactic acid bacteria in BP samples during fermentation. Vertical error bars correspond to standard deviations (n = 3). BP = bee-collected pollen.

The pH values of the BP samples were slightly acidic, ensuring an environment conducive to beneficial microbial activity while inhibiting harmful bacteria. Acidity, often measured as titratable acidity, reflects the concentration of organic acids produced, influencing flavor and preservation. During fermentation, the pH of all of the samples decreased as the lactic acid bacteria produced acids, while the acidity increased. Figure 1 shows the dynamics of bacterial growth during the 72 h of fermentation in BP samples, where the exponential growth phase can be observed. After 20 h, the bacterial growth entered the stationary phase.

Initially, the introduction of lactic acid bacteria triggers the fermentation process; then, the bacterial growth follows a typical pattern: a lag phase where bacteria adapt to the new environment, followed by a log phase characterized by exponential growth, and finally a stationary phase where growth stabilizes. As bacteria proliferate, they metabolize sugars in the BP, producing lactic acid. This accumulation of lactic acid causes a gradual decrease in pH, creating an acidic environment. The rate of pH drops, and the increase in acidity depends on factors like the bacterial strain, BP composition, and fermentation conditions. 

### 3.3. Effect of Fermentation on BP Nutritional Composition 

#### 3.3.1. Free Sugars

The free sugar contents of the BP and FBP samples were determined by HPLC/IR, and the results are shown in Table 3. The highest values were obtained for fructose and glucose, these being the majority of the free sugars in BP, whereas turanose and maltose were found in small quantities.

The addition, by bees, of nectar to the BP that they are going to deposit in the honeycomb cells has a major impact on the carbohydrate composition [53]. The fructose/glucose ratio for the BP samples was between 1.43 and 2.00, and for FBP samples it was between 1.48 and 3.09. Compared to the free sugar contents identified in the BP, a slight decrease in the carbohydrate content was found in the fermented samples, due to the fermentation process—a result also reported by other authors [54,55,56]. These changes can be explained by the fermentation of carbohydrates by probiotic microorganisms. These sugars do not represent the total carbohydrates from BP samples, but only the free sugars, most likely linked in glycosidic form. 

#### 3.3.2. Energy Value of BP

The total carbohydrate content and the nutritional value of BP samples can be visualized in Table 4. Protein is an extremely important parameter for the quality of BP, as well as for its energy value.

The results show that the amount of water in the BP samples before and after fermentation was similar, with no significant differences being recorded. The water content of the raw samples obtained directly from the beekeepers was influenced to a greater extent by the climatic conditions from the moment of harvesting.

The ash quantity reflects the total mineral content in BP samples and varies depending on the botanical and geographical origin of the BP, but there were no significant differences between the fermented and non-fermented samples.

Protein is an extremely important parameter for the quality of BP. A variation in the protein value of the BP samples was observed, between 17.15% in sample BP4 (*Salix* sp., *Prunus* sp.) and 23.61% in sample BP5 (*Plantago* sp., *Echium vulgare*). Regarding the FBP samples, a protein variation between 16.52% (BP4) and 22.21% (BP5) was observed. These variations between samples can be explained by the different botanical origins of the samples. In all samples, the average protein content decreased following the fermentation process, from 21.16% to 19.50%.

There were no significant differences in the percentage of lipids between the BP and FBP samples. The values obtained for BP varied between 4.11% (BP3—Salicaceae-*Salix* sp.) and 7.83 (BP2—Brassicaceae-*Brassica* sp.), with an average of 5.81%, while the variation in the case of FBP was between 4.00 and 7.65%, with an average of 5.56%. A slight decrease in total lipid values was found in all samples except for BP3 (Salicaceae-*Salix* sp). The lipid content of the natural pasture samples analyzed varied between 4.89 and 14.74%.

Campos et al. [6] linked the presence of carbohydrates, which are the primary constituents of pollen, with polysaccharides like starch and cell wall material, with values reaching up to 55%.

BP’s high nutritional content—which includes proteins, fats, and a substantial amount of carbohydrates—highlights its significance for bees and as a possible dietary supplement for humans. It is a complicated and advantageous dietary source because of its varied composition, which is impacted by the botanical sources. The FBP had an average energy value of 429.12 kcal/100 g, emphasizing that BP is a rich source of nutrients.

### 3.4. Free Amino Acid Contents 

**Figure 2:**
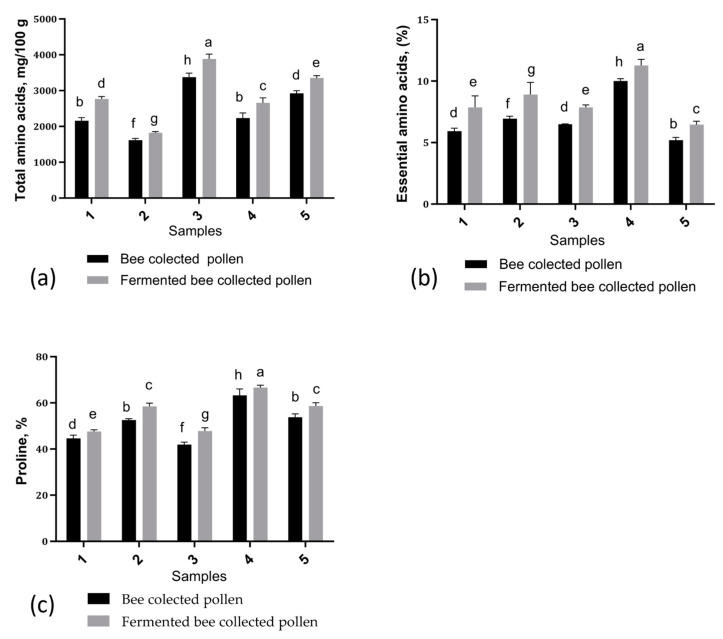
Amino acid profile of the samples analyzed before and after fermentation: (**a**) Total amino acid contents from BP samples before and after fermentation. (**b**) Contents of essential amino acids from BP samples before and after fermentation. (**c**) Proline content of BP samples before and after fermentation. BP = bee-collected pollen, FBP = fermented bee-collected pollen. Vertical error bars correspond to standard deviations (n = 3). Different letters represent significant differences (*p* < 0.05).

The profile of free amino acids in the BP samples before and after fermentation was analyzed by the LC-MS method. A total of 28 free amino acids were identified and quantified. The results can be seen in Figure 2 and Table 5.

The FBP samples had higher contents of free amino acids than the corresponding BP samples, which proves that hydrolysis of proteins took place during the fermentation process and, thus, although the total protein content decreased, they were transformed into amino acids following proteolysis. 

The highest value for total amino acids was identified in sample BP3 (3406.37 mg/100 g), and the lowest in sample BP2 (1598.19 mg/100 g). The values in the case of fermented BP were between 1696.83 and 3429.85 mg/100 g. The total amino acid contents of the BP samples varied depending on the floral origin. The nine essential amino acids (i.e., threonine, methionine, lysine, histidine, valine, tryptophan, leucine, phenylalanine, and isoleucine) were identified in the analyzed samples. Among all of the 28 amino acids identified, proline was identified in the largest amounts. In all analyzed samples, after fermentation, an increase in the value of total amino acids was observed.

### 3.5. Effect of Fermentation on Polyphenolic Profile

**Figure 3:**
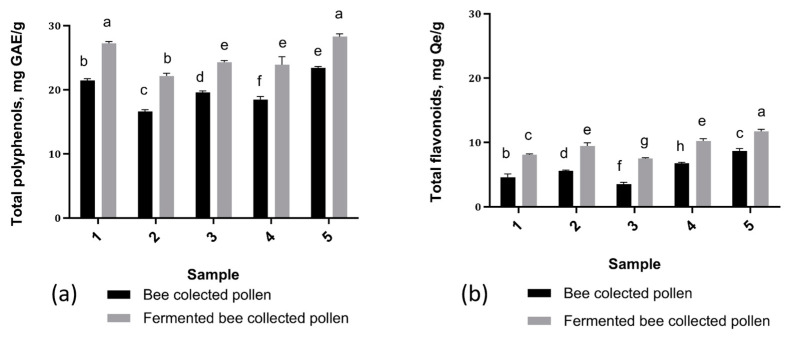
The total content of polyphenols and flavonoids (**a**) The total polyphenolic contents of BP and FBP samples. (**b**) Total flavonoid contents of BP and FBP samples. Vertical error bars correspond to standard deviations (n = 3). Different letters represent significant differences (*p* < 0.05).

Polyphenols and flavonoids represent significant groups of substances with different bioactive properties, and in the present work their total contents were quantified by spectrophotometric methods. Their variation in unfermented and fermented BP samples can be seen in Figure 3. 

The contents of total polyphenols ranged between 16.63 ± 0.20 mg GAE/g (BP2) and 23.44 ± 0.15 mg GAE/g (BP5), and a significant increase was observed in BP samples after fermentation, where the values varied between 22.18 ± 0.12 mg GAE/g (BP2) and 28.30 ± 0.29 mg GAE/g (BP5). Also, in the case of total flavonoids, the obtained values were lower in BP samples, where they varied between 3.51 ± 0.22 mg Qe/g (BP3) and 8.65 ± 0.30 mg Qe/g (BP5), compared to FBP, where the values were 7.50 ± 0.10 mg Qe/g (BP3) and 11.74 ± 0.23 mg Qe/g (BP5). Samples BP5 and FBP5 had the highest values.. In all of the analyzed samples, an increase in the total polyphenol and flavonoid values was observed after fermentation.

Furthermore, the variation in individual polyphenolic compounds in samples before and after fermentation is shown in Table 6. Identification of phenolic compounds from BP and FBP samples was carried out by the LC/MS method based on the main transition from the MS spectra of the substance.

The obtained phenolic profiles consisted mainly of flavonoids, but phenolic acids were also present. Caffeic, chlorogenic, and *trans-p*-coumaric acids were identified in variable amounts in all BP samples. In samples BP3 and BP4, which had pollen from *Salix* sp., salicylic acid was identified. Ellagic acid was identified in the largest amounts in the samples BP4 and FBP4. Flavanol kaempferol was identified in high amounts in sample BP5, with a value of 234.78 ± 0.14 µg/g in unfermented samples and 336.55 ± 0.31 µg/g in fermented samples. Other flavonoids, such as quercetin, quercetin-3-*O*-rutinoside, and luteolin, were identified in high quantities in the analyzed samples. Among the glycosides, rutin (quercetin-3-*O*-rutinoside) was identified in all samples at high concentrations. After the fermentation process in all samples, an increase in flavonoids and phenolic acids could be observed, except for chlorogenic acid. 

### 3.6. Impact of Fermentation on Antioxidant Activity

**Figure 4:**
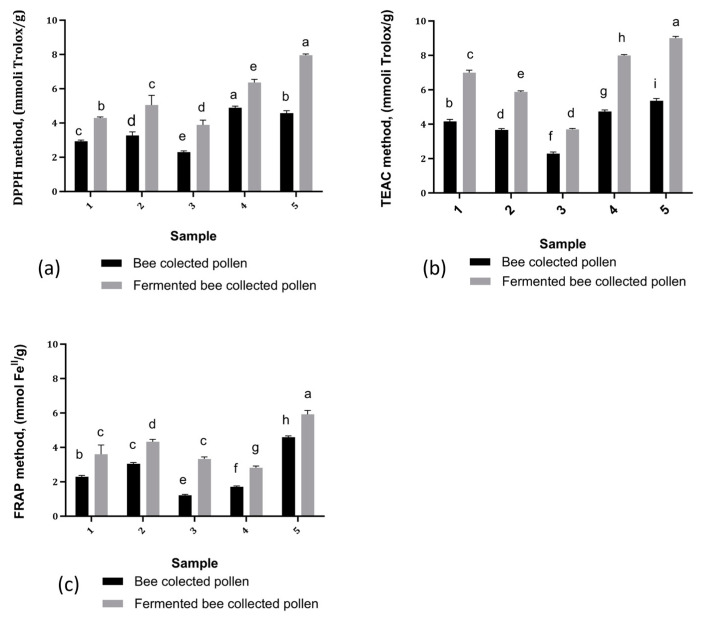
Antioxidant activity of BP and FBP samples tested using different methods: (**a**) The results regarding the antioxidant activity tested by DPPH method; (**b**) The results regarding the antioxidant activity tested by TEAC method; (**c**) The results regarding the antioxidant activity tested by FRAP method. Vertical error bars correspond to standard deviations (n = 3). Different letters represent significant differences (*p* < 0.05).

The antioxidant activity of the BP samples before and after fermentation was evaluated by DPPH, ABTS, and FRAP tests. The obtained results are presented in Figure 4.

All tested samples exhibited good antioxidant activity. However, among the three methods evaluated, the highest efficiency was against ABTS free radicals; the results obtained for BP samples were between 2.01 ± 0.16 mmol Trolox/g (BP3) and 5.36 ± 0.10 mmol Trolox/g (BP5), while for FBP the values were between 3.71 ± 0.0 mmol Trolox/g (FBP3) and 9.01 ± 0.15 mmol Trolox/g (FBP5). For the DPPH method, the results obtained for non-fermented BP ranged between 2.30 ± 0.09 (BP3) and 4.89 ± 0.12 mmol Trolox/g (BP4), and for FBP the lowest value was 3.88 ± 0.10 mmol Trolox/g (BP3 TR) and the highest was 7.96 ± 0.13 mmol Trolox/g (BP5). 

The weakest antioxidant effect was observed with the ferric reduction power method, where the antioxidant capacity for the unfermented samples varied between 1.22 ± 0.07 mmol/g Fe^II^ (BP3) and 4.60 ± 0.13 mmol/g Fe^II^ (BP5), and between 3.33 ± 0.21 mmol/g Fe^II^ (FBP1) and 5.92 ± 0.11 mmol/g Fe^II^ (FBP5) for the fermented ones. Regarding the antioxidant assays, the samples demonstrating the highest antioxidant activity were predominantly multifloral, containing *Plantaginaceae*, *Boraginaceae*, *Brasicaceae*, *Asteraceae*, *Sacicaceae*, and *Fabaceae* pollen.

Generally, the fermented samples showed a higher free radical scavenging activity compared to the non-fermented samples in the case of all tested methods, indicating that the fermentation seemed to have positively affected the antioxidant activity of the samples.

### 3.7. Impact of Fermentation on Antimicrobial Activity

The antimicrobial activity of the BP samples was first determined using the disk diffusion method against a variety of Gram-positive and Gram-negative bacterial strains, including *Staphylococcus aureus Enterococcus faecalis*, *Escherichia coli*, and *Pseudomonas aeruginosa*, along with the yeast *Candida albicans*. All examined bacteria showed good antimicrobial activity. The sizes of the inhibition zones are specified below in Table 7. 

Although the BP extracts had activity against both Gram-positive and Gram-negative bacteria, the Gram-positive ones were more sensitive, and the inhibition diameters obtained were larger. *E. faecalis* was the most sensitive to the action of the BP extracts, the best inhibition diameter being observed for the FBP sample (FBP4), with a value of 29.58 ± 0.25 mm. In the case of the unfermented samples, the BP1 sample also had the best effect against *E. faecalis*, with the inhibition diameter measuring 20.90 ± 0.11 mm. Among the Gram-negative strains, *E. coli* was more sensitive than *P. aeruginosa* to the action of the BP extracts, with the highest inhibition diameter being obtained for the FBP5 sample, at 27.11 ± 0.73 mm. When it comes to non-fermented samples, the equivalent sample BP5 had the best effect, with an inhibition diameter of 16.21 ± 0.78 mm. Regarding the effect of the BP on *C. albicans*, sample FBP5 had the best effect, where the resulting inhibition diameter measured 15.57 ± 0.17 mm. Among the non-fermented samples, BP5 also had the best effect, with a value of 10.65 ± 0.23 mm. Compared to samples from non-fermented BP, the fermented samples exhibited stronger antibacterial activity against all strains of microbes.

The minimum concentration required to inhibit the growth of a specific microorganism is referred to as the minimum inhibitory concentration (MIC) of the BP and FBP extracts. The serial microdilution technique was used to determine each sample’s minimum inhibitory concentration (MIC), and the findings are shown in Table 8. 

The findings obtained in this study indicated that the MIC values ranged from 0.39 to 25 mg/mL. The results obtained were in line with the inhibition diameter measurements, with Gram-positive bacteria being more sensitive than Gram-negative ones, so lower dosages were required to inhibit their development. Also, in this analysis, the fermented BP samples had better results, with lower MIC values than the non-fermented BP samples. The presence of specific antimicrobial compounds in the BP extracts, such as phenolic compounds or flavonoids, can significantly influence these values.

### 3.8. Statistical Analysis

**Figure 5:**
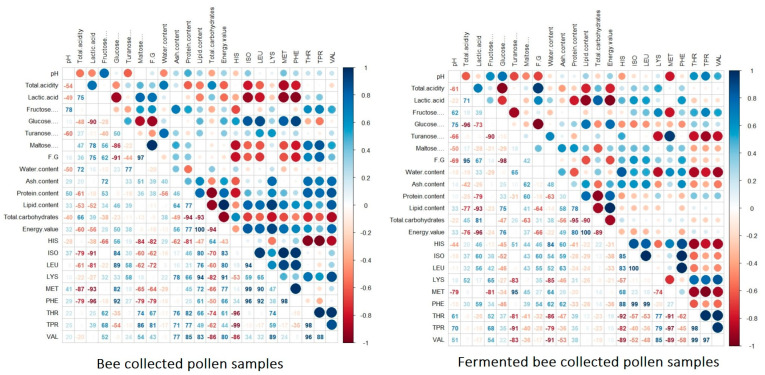
Correlograms showing graphical relationships among the evaluated nutritional values and essential amino acids of the BP (**left**) and FBP (**right**) samples. Correlation coefficients between the evaluated characteristics are displayed in red and blue. A darker hue and increased circle size represent a strong correlation, whereas a lighter hue and smaller circle size represent a weak correlation among the compounds.

According to the correlation matrix (Figure 5) and BP samples, the total acidity content showed a strong negative correlation with several essential amino acids, particularly ISO (*r* = −0.79), LEU (*r* = −0.61), MET (*r* = −0.87), and PHE (*r* = −0.79). Regarding lactic acid, a strong negative correlation with the glucose content and a strong positive correlation with maltose (*r* = −0.78) and the fructose/glucose ratio (*r* = −0.75) were noticed. Also, a strong negative correlation between ISO (*r* = −0.91), LEU (*r* = −0.81), MET (*r* = −0.93), and PHE (*r* = −0.96) was been, as in the case of total acidity. Glucose content was found to have a negative significant correlation with the maltose level (*r* = −0.86) and fructose/glucose ratio (*r* = −0.91), as well as a moderate negative association with TPR. Conversely, a strong positive correlation of glucose content with ISO (*r* = 0.84), LEU (*r* = 0.89), MET (*r* = 0.82), and PHE (*r* = 0.92) was noticed. The level of maltose was found to have a strong positive correlation with the fructose/glucose ratio (*r* = 0.97), as well as with the amino acids THR (*r* = 0.74) and TPR (*r* = 0.86). Conversely, a strong and negative correlation was observed with HIS (*r* = −0.84), ISO, LEU (*r* = −0.60), MET (*r* = −0.65), and PHE (*r* = −0.79). Regarding the essential amino acids, the water content presented a negative and weak association with the most of them. The protein content was negatively and strongly associated with total carbohydrates (*r* = −0.94) and HIS (*r* = −0.81). Conversely, a strong and positive association was observed with the individual essential amino acids, except for HIS, which had a negative and moderate association. The lipid content was found to have a positive and significant correlation with the energy value and the individual amino acids, as shown by the darker hue in the importance scores. The same positive and significant trend was shown in the case of energy value. Conversely, the total carbohydrates showed a strong and negative association with the essential amino acids. 

Regarding the fermented BP samples, significant differences were observed compared with the BP samples. Thus, the pH showed a positive and significant association with fructose and glucose, whereas a strong and negative association was shown with turanose and maltose. Intermediate association was recorded with the nutritional values and essential amino acids (i.e., MET; *r* = −0.79). The total acidity showed a strong and significant negative association with the glucose content (*r* = −0.96), lipid content (*r* = −0.77), and energy value (*r* = −0.76). The lactic acid content showed a strong and negative association with the levels of glucose (*r* = −0.73), protein (*r* = −0.79), lipids (*r* = −0.93), and energy value (*r* = −0.96), whereas a moderate positive association with HIS, ISO, LEU, and PHO was recorded. Regarding the fructose content, a strong negative correlation with turanose (*r* = −0.90) and MET (*r* = −0.81) was shown, whereas little to no association with the nutritional values was found. Also, a moderate and significant association with the essential amino acids was shown. Conversely, a strong and negative association of glucose with the fructose/glucose ratio (*r* = −0.98) was revealed, along with a moderate and negative correlation with most of the essential amino acids. The turanose content was strongly and negatively associated with several essential amino acids, including LYS (*r* = −0.83), THR (*r* = −0.81), TPR (*r* = −0.91), and VAL (*r* = −0.83). However, no association with the lipid content, energy value, ISO, LEU, or PHE was recorded. Regarding the water content, similar positive and negative correlations with the essential amino acids were shown as in the case of turanose. The protein and lipid contents were revealed to have a strong and positive association with the energy value and a strong negative association with the total carbohydrates. Furthermore, moderate and intermediate correlations of ash, protein lipids, and total carbohydrates with the essential amino acids were shown. 

**Figure 6:**
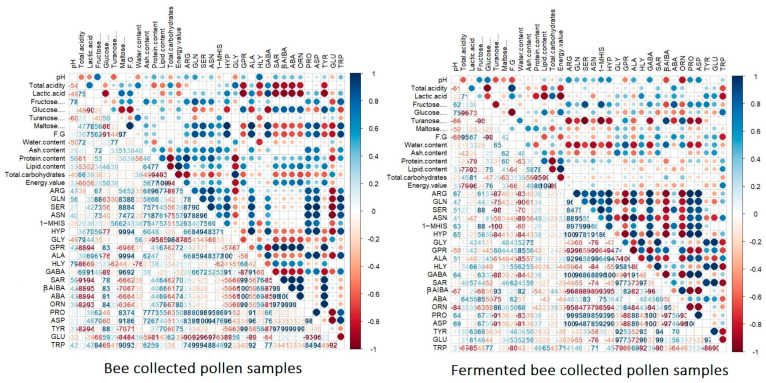
Correlograms showing graphical relationships among the evaluated nutritional values and non-essential amino acids of the BP (**left**) and FBP (**right**) samples. Positive and negative correlations between the evaluated characteristics are displayed in red and blue, respectively. A darker hue represents a strong correlation, whereas a lighter hue represents a weak correlation among the compounds.

According to the second correlation matrix (Figure 6) and non-essential amino acids, significant differences were observed between the BP and FBP samples. Thus, the pH value was shown to have a negative and significant association with total acidity, lactic acid, and turanose. Also, significant and positive correlations were recorded with the nutritional value of the BP samples. Regarding the total acidity and lactic acid content, significant negative associations with the energy value and non-essential amino acids—namely, GPR (*r* = −0.88), HLY (*r* = −0.86), SAR (*r* = −0.91), βAIBA (*r* = −0.88), AB (*r* = −0.88), ORN (*r* = −0.82), and TYR (*r* = −0.82)—were recorded, as seen by the darker red hue according to the importance scores, whereas significant positive correlations were shown with the maltose levels, fructose/glucose ratio, and total carbohydrates. Little to no association was recorded with the remaining non-essential amino acids, as shown by the light hue on the correlation matrix.

The maltose content and fructose/glucose ratio were shown to have similar significant positive correlations with ARG, GLN, SER, ASN, HYP, ALA, GABA, PRO, ASP, and TRP. Conversely, negative significant correlations were observed with the remaining non-essential amino acids, namely, GPR, SAR, βAIBA, ABA, ORN, TYR, and GLU. The protein content, lipid content, and energy value presented similar and significant positive associations with the energy value and ARG, GLN, SER, ASN, 1-MHIS, SAR, βAIBA, ABA, ORN, PRO, ASP, and TYR. The total carbohydrates presented significant and negative associations with almost all non-essential amino acids, except for GLY (*r* = 0.98), which showed a strong positive correlation. 

Regarding the fermented BP samples, the pH presented significant positive association with fructose (*r* = 0.62), glucose (*r* = 0.75), ARG (*r* = 0.67), 1-MHIS (*r* = 0.63), HYP (*r* = 0.65), GABA (*r* = 0.64), PRO (*r* = 0.64), and ASP (*r* = 0.69). Conversely, negative and significant associations with GPR (*r* = −0.50), βAIBA (*r* = −0.67), and ORN (*r* = −0.84) were recorded. The total acidity was shown to have a positive association with the fructose/glucose ratio (*r* = 0.95) and a moderate association with SAR and ABA. Lactic acid presented similar significant and negative associations as in the case of total acidity, with glucose (*r* = −0.73), protein (*r* = −0.79), and lipid levels (*r* = −0.93), energy value (*r* = −0.96), and TRP (*r* = −0.85). Fructose presented significant positive correlations with SER (*r* = 0.88), 1-MHIS (*r* = 0.88), and ABA (*r* = 0.85), as well as intermediate associations with ARG, GLN, GABA, PRO, ASP, and TYR. The protein and lipid contents were shown to have significant and positive associations with the energy value (*r* = 0.80; *r =* 100), as well as strong and negative associations with the total carbohydrates (*r* = −0.95; *r =* −0.90). Insignificant associations with the non-essential amino acids were found. A similar trend was observed regarding the correlation of energy values with the non-essential amino acids. 

**Figure 7:**
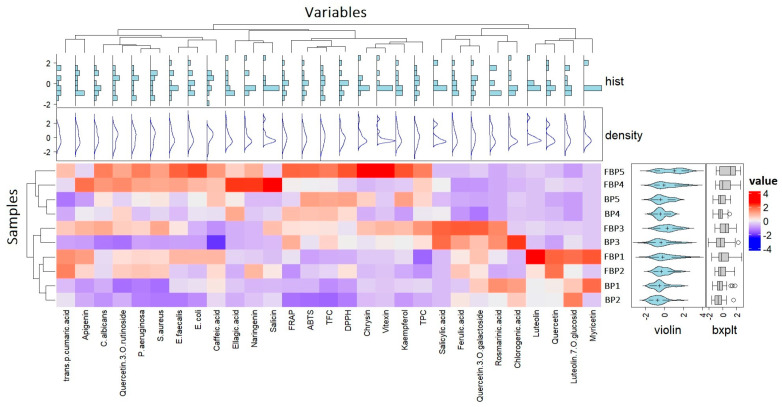
Hierarchical clustering and heatmap visualization of polyphenolic profile, antioxidant activities, and antimicrobial activities of fermented and non-fermented BP samples. Columns indicate the identified phenolic compounds, antioxidant activities, and antimicrobial activities, and rows indicate the BP and FBP samples. Cells are colored based on the values of identified compounds and bioactivities, where red represents a strong positive correlation and blue a strong negative correlation.

Hierarchical clustering (HCA) and heat mapping were used to better visualize the similarities and differences in polyphenolic profile, antioxidant activities, and antimicrobial activities between the BP and FBP samples (Figure 7).

The HCA revealed an unambiguous discrimination between the biologically active compounds and BP samples seen via the different cluster positions of the fermented BP samples. The first cluster revealed the grouping of the FBP samples (i.e., FBP4–FBP5), highlighting the FBP5 sample, which had increased levels of polyphenolics—particularly chrysin, kaempferol, and vitexin—and the highest antioxidant activity against ABTS. Furthermore, increased antimicrobial activity was observed against Gram-positive bacteria, Gram-negative bacteria (i.e., *E. coli*), and *C. albicans*, which may have been due to the accumulation of phenolic compounds, particularly kaempferol and quercetin-3−O−rutinoside. This sample was closely followed by FBP4, which presented similar antimicrobial activities to FBP5. Conversely, increased accumulation of apigenin, ellagic acid, naringenin, and salicin was observed. Subsequently, the following sub-cluster comprised the BP samples BP4 and BP5, which were highlighted by their similar total phenolic compounds and antioxidant activities against DPPH and ABTS. The second cluster comprised the fermented (i.e., FBP3) and non-fermented (i.e., BP3) samples that presented the highest accumulation of salicylic acid and similar values for rosmarinic acid. Conversely, compared to the fermented samples, BP3 accumulated increased levels of chlorogenic acid. The subsequent sub-cluster highlighted the FBP samples FBP1 and FBP2, which accumulated similar and significant levels of quercetin and *trans*-*p*-coumaric acid, whereas, contrary to FBP2, increased accumulation of luteolin was observed in FBP1. Furthermore, similar and insignificant activity against the Gram-negative bacteria was observed. The last sub-cluster emphasized the BP1 and BP2 samples, which presented similar and insignificant antimicrobial activity against Gram-negative bacteria (i.e., *P. aeruginosa*) and *C. albicans.*

## 4. Discussion

BP is already known for its rich nutritional contents, but the bioavailability of these nutrients can be limited. The existing studies in the literature show that fermentation with these specific strains can break down complex nutrients into more bioavailable forms, enhancing the nutritional profile of BP [16,28,33,35]. In this study, five BP samples were fermented using *Lactobacillus plantarum* ATCC 8014, *Apilactobacillus kunkeei* ATCC 700308, and *Lactobacillus acidophilus* ATCC 700396. The mix of LAB for the fermentation of BP was chosen based on the unique properties and synergistic effects of these bacteria. *L. plantarum* is known for its versatility in fermenting various substrates [57,58]. It can adapt to different environments, making it highly effective for fermenting bee pollen, which has a complex composition [31]. According to data from the literature [59], this strain can enhance the bioavailability of phenolic compounds in the pollen, contributing to the overall increase in nutritional and bioactive properties. *L. acidophilus* is a well-known probiotic that is beneficial for gut health [59]; incorporating it into fermented BP can add probiotic properties to the final product, making it more beneficial for consumption. It is effective in producing lactic acid, which can help in preserving the BP and maintaining a stable pH during fermentation [25]. *A. kunkeei* is commonly found in bee products and is well adapted to the bee environment [60]. Its inclusion ensures a more natural and bee-friendly fermentation process because it is particularly adapted to the unique composition of bee-derived substrates, potentially making the fermentation process more efficient and tailored to the specific characteristics of the BP [61]. This bacterium prefers fructose over glucose, which is beneficial since BP and related products are rich in fructose [62]. This can lead to a more targeted and efficient fermentation process. All samples were analyzed before and after the fermentation process, evaluating the nutritional composition, amino acid content, and biologically active compounds such as polyphenols and flavonoids, but also the antioxidant and antimicrobial activities.

The development of lactic acid bacteria, along with the pH, acidity, and lactic acid production, were monitored during BP fermentation. As the LAB proliferated, they metabolized sugars from the BP samples, producing lactic acid, which caused a gradual decrease in pH, creating an acidic environment. The rate of pH drops and acidity increases depended on factors like bacterial strain, BP composition, and fermentation conditions. These results were also observed in other studies that considered BP fermentation using LAB [15,31,40,43]. 

Among the carbohydrates identified in BP, fructose represented the majority, followed by glucose. Lactic fermentation significantly impacts BP’s sugar contents, primarily reducing the overall sugar content as fermenting bacteria metabolize sugars for energy. This process transforms simple sugars into organic acids, notably lactic acid, enhancing the BP’s nutritional profile. In all BP samples after fermentation, the amounts of fructose and glucose decreased. The fructose/glucose ratio for the BP samples was between 1.43 and 2.00, while for FBP samples it was between 1.48 and 3.09. Sample BP5 had the highest amount of fructose, at 20.12 ± 0.12%, while sample BP1 had the highest amount of glucose, at 12.14 ± 0.22%. Meanwhile, in the fermented samples, the highest values for fructose were recorded in sample FBP1, at 16.60 ± 0.21%, while for glucose the largest quantity was found in FBP2, at 9.61 ± 0.18%. The results obtained for BP were similar to those obtained by other authors. Szczesna [53] reported the presence of fructose in the highest amounts, followed by glucose. Furthermore, Yan et al. [12] and Hsu et al. [63] also reported higher contents of fructose compared to glucose in BP samples, but also significant decreases after the BP fermentation process with different types of microorganisms. The variation in the values obtained for fructose and glucose in the analyzed samples is normal; the sugar composition of BP can vary significantly depending on its botanical origin, with samples from different regions showing considerable differences in sugar contents and types of sugars present [6,64]. 

The lipid content in BP is noted for its range of fatty acids, including omega-3 and omega-6, which are vital for health [9]. The lipid content in BP varied from 4.11 ± 0.24% to 7.58 ± 0.18%, and from 4.19 ± 0.21% to 7.48 ± 0.31% in FBP, which is in line with values reported by other authors [63]. These lipids contribute significantly to the energy value of BP, are an important component of its nutritional profile, and have relatively constant values after the fermentation process. 

BP is recognized for its high energy value due to its rich contents of carbohydrates, proteins, and lipids. The mean energy value reported is around 375 kcal/100 g, indicating its potential as a substantial dietary supplement [65].

BP is a high-protein source, and its quality is often evaluated as an index of nutritional value [66]. Moreover, BP provides essential amino acids that are crucial for both bees and humans, with quantities in certain samples meeting or exceeding the minimum required levels [63]. In this study, the average protein content for the unfermented BP samples was equal to 19.50 ± 0.15%, while in the FBP a slight increase occurred, with the samples having an average of 21.16 ± 0.18%. The variation in the values reported by other authors for the protein content of BP is large, with the literature mentioning that BP naturally contains about 10–52% protein [42,65] and is rich in essential amino acids. After undergoing fermentation, the nutrient availability changes, and this process seems to enrich the protein content and make the BP more nutritious, especially in terms of its protein and amino acid composition [67]. 

Following the LC/MS analysis performed here, a total of 28 free amino acids were identified and quantified in the analyzed samples, and it seemed that during lactic fermentation the amino acid profile in the BP underwent significant changes. The fermentation process led to an increase in free amino acids in all five samples. 

The amino acid profiles of the five BP samples tested (Table 5) reflect the high quality of the proteins, proving the samples to be significant sources of complete proteins with a good balance of essential amino acids. In addition, the contents of essential amino acids were highlighted (Figure 2b), and the results showed that the fermentation led to an increase in the amounts of essential amino acids. The most abundant essential amino acids in all samples were leucine, methionine, histidine, valine, and lysine. Among these, leucine was identified at the largest amount in samples BP5 (148.50 ± 0.28 mg/100 g) and FBP (5169.4 ± 0.25 mg/100 g).

Moreover, all analyzed BP samples contained conditionally essential amino acids such as arginine, glutamine, tyrosine, glycine, serine, and proline. These amino acids are usually synthesized by the body in sufficient quantities, but under certain conditions, such as severe stress or disease, they can become essential [68]. Of these, proline was found in the largest quantity in the analyzed BP, and its variation can be observed in Figure 2c. Proline is essential for collagen synthesis, contributes to tissue repair and regeneration, and supports immune function and intestinal integrity. It participates in protein metabolism and ammonia elimination, regulates apoptosis for cellular balance, and protects cells against oxidative stress thanks to its antioxidant properties. This result is also supported by the findings of Domıinguez-Valhondo et al. [69]. Proline and glutamic acid levels are linked to the quality of BP [6,70].

The obtained results are also supported by other studies in the literature. Yan et al. [12] analyzed amino acids in rape bee pollen (*Brassica campestris* L.) BP under fermentation, revealing proline as the most abundant, and all essential amino acids were present. Post-fermentation, the amino acids increased significantly, indicating enhanced protein hydrolysis. Kieliszek et al. [71] observed similar amino acid elevation in fermented BP, likely due to microbial degradation. Degrandi-Hoffman et al. [72] found leucine to be the most prevalent essential amino acid in BP and bee bread, with higher total essential amino acids in bee bread. Darwish et al. [67] identified 17 amino acids in BP, with notable levels of essential amino acids such as leucine, lysine, and phenylalanine. Fermentation induced changes in protein quantity and amino acid content, enhancing the nutritional quality. Amino acid variation in BP is influenced by microbial activity and fermentation conditions, with botanical origin also playing a significant role [73]. 

To highlight the effect of fermentation on the phenolic compounds in BP, the total amounts of polyphenols and flavonoids were determined before and after fermentation. The data obtained indicate that the phenolic profile of BP was positively impacted by fermentation. The results of the statistical analysis demonstrated that the polyphenol contents of the BP samples before and after fermentation differed significantly. Regarding the contents of total flavonoids, the obtained results showed that fermentation had a positive effect on their amount: in the case of all samples, a higher value was identified after fermentation. The variation in the amounts of total polyphenols and flavonoids in the BP samples was due to their different botanical origins [3,32,46], as has also been shown in other studies. 

Kaškonienė et al. [28] found that fermentation boosted the flavonoid content in bee pollen (BP), with levels increasing from 2.6–5.7 mg/g to 6.0–9.3 mg/g. This 1.6–2.4-fold rise was observed in all fermented BP samples, suggesting the involvement of native microflora. Another study [35] reported a TPC increase ranging from 12.0% to 89.1%, influenced by the fermentation type and botanical origin of the pollen

Furthermore, LC/MS analysis also revealed chemical changes in non-fermented vs. fermented samples. The results revealed a complex heterogeneous mixture of phenolic compounds in all samples and represent a fingerprint of BP potentially serving as an analytical tool to ascertain the botanical provenance of the pollen [74,75].

Among the seven phenolic acids identified, caffeic acid, *trans-p*-coumaric acid, and ellagic acid were predominant, with samples BP4 and BP5 exhibiting the highest concentrations, alongside a spectrum of thirteen flavonoids. Notably, the flavones quercetin and kaempferol were present in all samples, with BP1 and BP5 exhibiting elevated levels. Glycosidic flavonoids, including quercetin-3-*O*-rutinoside, quercetin-3-*O*-galactoside, and luteolin-7-O-glucoside, were detected, alongside apigenin, chrysene, luteolin, naringenin, and vitexin, albeit in lesser amounts (see Table 6). Among the glycosides, quercetin-3-*O*-rutinoside was identified at high concentrations in all samples. In BP, rutin is the most frequently identified glycoside, according to the literature [76]. The presence of rutin in BP is indicative of its biological and nutritional value, owing to its notable high antioxidant activity, as reported in [77,78], but also its antiviral, antibacterial, cytoprotective, anti-inflammatory, vasoactive, antitumor, antioxidant, cardioprotective, and antispasmodic activities. 

Consistent with the existing literature [3,7,79,80,81,82], phenolic compounds are often present in BP and other bee products at different concentrations according to the botanical and geographic origin, and they are responsible for the bioactive properties.

In all of the samples, variable increases in the contents of phenolic compounds were identified. Notably, regarding phenolic acid, the highest increase was identified in p-coumaric acid concentration. P-coumaric acid is a crucial component of sporopollenin, which forms the outer wall of pollen. This observation highlights the complex relationship between fermentation, the accessibility of compounds, and the intricate structure of pollen. This increase after fermentation was also observed by other authors [70].

Previous studies [14,76,83] have identified a range of phenolic compounds such as caffeic, chlorogenic, ferulic, coumaric, and gallic acids, along with flavonoids like quercetin and naringenin, in both BP and FBP. These studies elucidate the transformative influence of BP fermentation, underscoring a metabolic activity that fosters the synthesis of components akin to those found naturally in FBP and bee bread. The fermentation process manifests its effects predominantly by enhancing the diversity and intensity of biologically active substances, as evidenced by the concentration increase post-fermentation [30]. However, in this study, there was an exception, and a decrease in chlorogenic acid was observed after the fermentation process in all samples.

The dynamic interaction between LAB and phenolic compounds during fermentation plays a pivotal role in reshaping the chemical composition of bee-collected pollen, and sometimes the enzymatic activities of LAB break down complex compounds into simpler ones. This reduction can be attributed to the esterase activity of lactic acid bacteria, which hydrolyzes this particular compound [83,84] and was previously reported by Adaškevičiūtė et al. [76], who observed a significant decrease of up to 49.5% in chlorogenic acid content following bacterial fermentation, with variations in naringenin linked to the fermentation type and pollen source. Phenolic stability in Polish BP across studies suggests a regional influence. Fermentation induces structural and chemical changes in phenolic compounds, potentially altering their bioavailability and effects. Studies [14] have noted increased free phenolics and antioxidant activity in fermented BP. Lactic acid fermentation by Lactococcus lactis and Lactobacillus rhamnosus enhances total phenolics and antioxidant activities [85]. Kaškonienė et al. [28] showed that solid-state fermentation with *L. rhamnosus* GG elevated the total flavonoid content by 55–135%, emphasizing fermentation’s benefits on BP’s antioxidant activity.

The presence of phenolic acids, such as ferulic, caffeic, and p-coumaric acids, but also of flavanols, kaempferol, quercetin, glycosides, quercetin-3-*O*-rutinoside, quercetin-3-*O*-galactoside, and luteolin-7-O-glucoside in the composition of the samples, known for their antioxidant effects [21,74,85,86], contributes to the overall antioxidant capacity of pollen. These phenolic compounds, whether acting individually or in synergy, play a pivotal role in the health-enhancing qualities of pollen, rendering it a valuable dietary supplement.

Research findings indicate that solid-state lactic acid fermentation can lead to a substantial increase (1.4–2.3 times) in the radical scavenging activity of BP. This action can be explained by the presence of phenolic acids and flavonoids, known for their strong antioxidant properties [35]. Consequently, this fermentation not only enriches BP with health-beneficial compounds, but also elevates its antioxidant activity significantly, highlighting the valuable role of fermentation in enhancing the nutritional and functional characteristics of bee pollen. Although all three tested antioxidant techniques showed good antioxidant activity, the highest efficiency was against ABTS free radicals. The ABTS method is sensitive to both hydrophilic and lipophilic antioxidants [87]; therefore, it is possible that the antioxidant components of BP extracts are more effective in neutralizing ABTS radicals, possibly due to dual solubility or specific chemical affinity. Regarding the reducing power of FRAP, the opposite tendency was seen, which is further supported by additional research [88]. After fermentation, the radical scavenging activity increased by 36–87% in all natural BP samples (*p* < 0.05), although it increased by 15–49% in pasteurized samples. 

The antibacterial activity of the BP and FBP samples was strongly related to the bacteria used for the antibacterial tests. In the case of the disk diffusion method, the best inhibition diameters were obtained for *E. fecalis* and *S. aureus*, followed by *E. coli*, while *P. aeruginosa* showed the highest resistance to the BP extracts, indicating that Gram-positive bacteria were more sensitive than Gram-negative ones. Regarding the results obtained for the yeast *C. albicans*, the inhibition diameters were similar to those obtained for *P. aeruginosa*. Generally, the samples of FBP exhibited higher antimicrobial activity, obtaining larger diameters of inhibition compared to the non-fermented ones. Consequently, fermentation represents a potential avenue for augmenting the antimicrobial properties of bee pollen, thus expanding its potential applications. 

The MIC values obtained varied between 0.39 and 25 mg/mL depending on the type of microorganism and the BP sample. The samples BP5 and FBP5 had the best effects against the strains tested, with the MIC varying between 0.39 and 12.50 mg/mL, the most resistant being the *P. aeruginosa*. The sample BP5, which had the highest contents of polyphenols and flavonoids, but also of individual polyphenols, generally had the best antimicrobial effect, which indicates a link between the presence of these compounds and the improved antimicrobial action; this finding is also supported by other authors [35]. Pełka et al. [89] found higher antimicrobial potential in natural FBP compared to BP. Bee bread extracts showed inhibitory potential against *S. aureus* strains, while some pollen extracts lacked efficacy. At doses ranging from 2.5 to 5.0%, the majority of active extracts successfully stopped the growth of clinical isolates of *S. aureus*, including MRSA strains. In another study [35], FBP demonstrated increased antimicrobial activity (by 1.08–16.9 times) against *S. aureus* and *E. coli* post-fermentation. The authors noted fermentation’s favorable impact on BP’s biological properties, influenced by the botanical source and fermentation method. 

Other investigations have shown that fungi and yeasts are more resistant than bacteria to the effects of BP extracts, which is another point of emphasis in the literature [90,91].

It is important to interpret these values obtained for the antimicrobial activity with caution, as they are highly dependent on the specific conditions of this study, including the methods of extraction, concentration of the extracts, and specific strains of microorganisms used. Some strains of this bacterium might show no sensitivity to certain BP extracts, while others might be more sensitive. 

## 5. Conclusions

The findings of this study demonstrate that the fermentation of BP with *Lactobacillus plantarum, Lactobacillus acidophilus*, and *Apilactobacillus kunkeei* significantly enhances its nutritional and bioactive properties. The analysis revealed notable improvements in amino acid contents, polyphenolic profiles, and flavonoid levels post-fermentation, suggesting increases in the health-promoting compounds of BP. Furthermore, heat mapping and cluster analysis demonstrated increased antioxidant and antimicrobial activities in the fermented BP samples, particularly against Gram-positive and Gram-negative bacteria such as *E. coli*. This enhanced activity may be attributed to the accumulation of phenolic compounds, including ellagic acid, kaempferol, quercetin, and quercetin-3-*O*-rutinoside, during the fermentation process. Future research prospects include exploring the mechanistic pathways through which these *Lactobacillus* strains enhance the nutritional and bioactive properties of BP, investigating the scalability of the fermentation process, and assessing the feasibility of incorporating fermented BP into various functional food products. Additionally, clinical studies to evaluate the health benefits of fermented BP in human diets would provide concrete evidence for its functional properties and further validate its potential as a valuable dietary supplement.

## Figures and Tables

**Table 1:** Family and plant species of the BP from the analyzed samples.

Sample	Predominant Pollen (>45%) Family-Species	Secondary Pollen (16–45%) Family-Species	Important Minor Pollen (3–15%) Family-Species	Minor Pollen (<3%) Family-Species
BP1		*Fabaceae*, *Trifolium pratense* *Rosaceae*, *Prunus* sp.	*Asteraceae:* *Centaurea* sp. *Taraxacum* sp. *Achilea millefolium* *Bellis perennis* *Cirsium arvense* *Tiliaceae*, *Tilia* sp. *Brassicaceae*, *Brassica* sp. *Fabaceae*, *Onobrichys viicifolia*	*Plantaginaceae, Plantago* sp. *Rosaceae* *Apiaceae* *Asteraceae,* *Centaurea* sp. *Lamiaceae*
BP2	*Brassicaceae, Brassica* sp.	*Asteraceae*, *Centaurea* sp. *Centaurea cyanus* *Rosaceae, Rubus* sp.	*Fabaceae, Vicia* sp. *Asteraceae:* *Matricaria* sp. *Taraxacum* *Apiaceae*	*Boraginaceae, Phacelia tanacetifolia*
BP3	*Salicaceae, Salix* sp.	*Asteraceae,* *Taraxacum* sp.	*Fabaceae:* *Trifolium* sp. *Trifolium pratense*	*Rosaceae*
BP4		*Salicaceae, Salix.* sp. *Rosaceae, Prunus* sp.	*Asteraceae*, *Taraxacum* sp. *Fabaceae*, *Trifolium* sp.	
BP5		*Plantaginaceae*, *Plantago* sp. *Boraginaceae*, *Echium vulgare*	*Brassicaceae*, *Brassica* sp. *Asteraceae*, *Taraxacum* sp. *Salicaceae*, *Salix* sp. *Fabaceae*, *Trifolium* sp. *Apiaceae* *Lamiaceae* *Gramineae* *Rosaceae*	*Tiliaceae*, *Tilia* sp.

**Table 2:** pH, acidity, and lactic acid content of BP before and after fermentation.

Sample	pH		Total Acidity meq NaOH kg^−1^		Lactic Acid, %	
	BP	FBP	BP	FBP	BP	FBP
1	5.07 ± 0.10 ^a^	4.29± 0.11 ^b^	16.69 ± 0.14 ^b^	25.34± 0.16 ^a^	3.19 ± 0.06 ^b^	4.70 ± 0.13 ^a^
2	4.82 ± 0.23 ^a^	4.13± 0.19 ^b^	17.83 ± 0.93 ^b^	23.53± 0.21 ^a^	3.22 ± 0.11 ^b^	4.47 ± 0.08 ^a^
3	4.72 ± 0.17 ^a^	3.87± 0.15 ^b^	18.89 ± 0.56 ^b^	27.18± 0.25 ^a^	3.69 ± 0.13 ^b^	4.95 ± 0.17 ^a^
4	4.89 ± 0.21 ^a^	4.19± 0.24 ^b^	19.83 ± 0.77 ^b^	25.65± 0.44 ^a^	3.51 ± 0.07 ^b^	5.14 ± 0.19 ^a^
5	4.92 ± 0.33 ^a^	4.01± 0.21 ^b^	18.85 ± 0.23 ^b^	26.57± 0.31 ^a^	3.71 ± 0.15 ^b^	4.84 ± 0.22 ^a^
Average	4.88 ± 0.21	4.09 ± 0.18	18.41 ± 0.53	25.65 ± 0.27	3.46 ± 0.10	4.82 ± 0.16

BP = bee-collected pollen, FBP = fermented bee-collected pollen. Results represent the mean ± standard deviation of three independent determinations. Within the same column, different letters indicate significant differences (*p* < 0.05).

**Table 3:** Free sugar contents of BP before and after fermentation.

Sample	Fructose (%)		Glucose (%)		Turanose (%)		Maltose (%)		F/G	
	BP	FBP	BP	FBP	BP	FBP	BP	FBP	BP	FBP
1	19.10 ± 0.15 ^b^	16.60 ± 0.21 ^a^	10.21 ± 0.21 ^e^	8.50 ± 0.22 ^e^	0.75 ± 0.01 ^a,c^	0.65 ± 0.11 ^c^	0.49 ± 0.01 ^b^	0.39 ± 0.02 ^b^	1.87	1.95
2	17.34 ± 0.25 ^c^	14.230 ± 0.25 ^b^	12.14 ± 0.22 ^a^	9.61 ± 0.18 ^a^	1.13 ± 0.02 ^a^	1.00 ± 0.09 ^a^	0.48 ± 0.09 ^b^	0.44 ± 0.08 ^a^	1.43	1.48
3	16.20 ± 0.1 ^d^	14.42 ± 0.1 ^b^	8.11 ± 0.30 ^b^	4.90 ± 0.10 ^b^	0.90 ± 0.01 ^b^	0.99 ± 0.15 ^a^	0.52 ± 0.02 ^a^	0.42 ± 0.08 ^b^	2.00	2.94
4	18.81 ± 0.30 ^b^	16.31 ± 0.13 ^a^	9.53 ± 0.15 ^c^	6.82 ± 0.28 ^c^	0.94 ± 0.02 ^b^	0.86 ± 0.01 ^b^	0.52 ± 0.10 ^a^	0.38 ± 0.02 ^b^	1.97	2.39
5	20.12 ± 0.12 ^a^	15.98 ± 0.22 ^a^	7.10 ± 0.21 ^d^	5.56 ± 0.24 ^d^	0.89 ± 0.02 ^b^	0.82 ± 0.02 ^b^	0.61 ± 0.04 ^a^	0.51 ± 0.03 ^a^	2.83	3.09
Average	18.31 ± 0.18	15.74 ± 0.20	9.41 ± 0.22	7.07 ± 0.20	0.92 ± 0.02	0.86 ± 0.08	0.52 ± 0.05	0.42 ± 0.05	1.94	2.22

BP = bee-collected pollen, FBP= fermented bee-collected pollen. Results represent the mean of three independent determinations. Within the same column, different letters indicate significant differences (*p* < 0.05).

**Table 4:** Nutritional value of BP before and after fermentation.

Sample	Water Content (%)		Ash Content (%)		Protein Content (%)		Lipid Content (%)		Total Carbohydrates (%)		Energy Value kcal/100 g	
	BP	FBP	BP	FBP	BP	FBP	BP	FBP	BP	FBP	BP	FBP
1	20.21 ± 0.51 ^c^	19.32 ± 0.12 ^c^	2.44 ± 0.12 ^b^	2.41 ± 0.10 ^b^	20.71 ± 0.10 ^b^	22.80 ± 0.18 ^b^	6.11 ± 0.21 ^d^	5.94 ± 0.14 ^c^	68.65 ± 0.26 ^b^	70.94 ± 0.31 ^b^	431.77 ± 0.57 ^d^	431.01 ± 0.46 ^d^
2	26.39 ± 0.40 ^a^	25.65 ± 0.17 ^d^	2.65 ± 0.15 ^c^	2.70 ± 0.12 ^a^	20.00 ± 0.22 ^b^	22.36 ± 0.17 ^b^	7.58 ± 0.18 ^a^	7.48 ± 0.31 ^a^	67.41 ± 0.29 ^b^	69.87 ± 0.36 ^b^	438.55 ± 0.51 ^a^	437.54 ± 0.43 ^a^
3	23.47 ± 0.31 ^b^	23.39 ± 0.23 ^b^	2.25 ± 0.11 ^d^	2.31 ± 0.12 ^b^	18.06 ± 0.18 ^c^	19.88 ± 0.21 ^c^	4.11 ± 0.24 ^b^	4.19 ± 0.21 ^b^	73.76 ± 0.25 ^c^	75.44 ± 0.21 ^c^	422.15 ± 0.42 ^b^	422.32 ± 0.36 ^b^
4	27.81 ± 0.11 ^a^	27.53 ± 0.17 ^a^	2.62 ± 0.14 ^c^	2.59 ± 0.22 ^b^	17.06 ± 0.09 ^d^	17.65 ± 0.24 ^d^	4.62 ± 0.16 ^c^	4.28 ± 0.11 ^b^	75.11 ± 0.29 ^a^	76.11 ± 0.24 ^a^	423.28 ± 0.31 ^b^	421.64 ± 0.37 ^b^
5	23.77 ± 0.25 ^b^	23.67 ± 0.13 ^b^	2.81 ± 0.10 ^a^	2.76 ± 0.11 ^a^	21.71 ± 0.15 ^a^	23.11 ± 0.12 ^a^	6.66 ± 0.20 ^d^	5.99 ± 0.12 ^c^	67.41 ± 0.36 ^b^	69.54 ± 0.33 ^b^	433.14 ± 0.29 ^c^	429.83 ± 0.39 ^c^
Average	24.33 ± 0.32	23.91 ± 0.16	2.55 ± 0.17	2.55 ± 0.13	19.50 ± 0.15	21.16 ± 0.18	5.81 ± 0.20	5.576 ± 0.18	70.46 ± 0.29	72.38 ± 0.29	429.77 ± 0.42	428.46 ± 0.40

BP = bee-collected pollen, FBP = fermented bee-collected pollen. Results represent the mean ± standard deviation of three independent determinations. Within the same column, different letters indicate significant differences (*p* < 0.05).

**Table 5:** Free amino acid contents of analyzed BP samples before and after fermentation.

Amino Acid	BP1	FBP1	BP2	FBP2	BP3	FBP3	BP4	FBP4	BP5	FBP5
	mg/100 g									
Essential amino acids										
HIS	46.10 ± 0.17	51.34 ± 0.21	56.21 ± 0.13	62.62 ± 0.20	0.41 ± 0.01	0.85 ± 0.01	37.1 ± 0.08	35.11 ± 0.06	56.00 ± 0.12	55.89 ± 0.13
ISO	17.60 ± 0.11	21.39 ± 0.01	5.23 ± 0.01	6.89 ± 0.01	8.27 ± 0.03	5.14 ± 0.02	9.3 ± 0.03	11.54 ± 0.05	31.30 ± 0.06	35.58 ± 0.08
LEU	34.60 ± 0.13	68.23 ± 0.22	11.28 ± 0.03	12.37 ± 0.02	13.23 ± 0.06	28.65 ± 0.07	45.8 ± 0.11	45.12 ± 0.10	148.50 ± 0.28	169.4 ± 0.25
LYS	28.80 ± 0.12	49.65 ± 0.01	20.14 ± 0.03	26.25 ± 0.08	44.25 ± 0.12	46.35 ± 0.09	7.3 ± 0.01	28.11 ± 0.09	19.80 ± 0.07	32.35 ± 0.06
MET	41.25 ± 0.17	45.12 ± 0.11	2.09 ± 0.01	2.35 ± 0.01	5.59 ± 0.02	6.81 ± 0.01	82.6 ± 0.13	85.69 ± 0.15	55.60 ± 0.11	68.65 ± 0.13
PHE	28.00 ± 0.11	32.58 ± 0.08	10.46 ± 0.02	11.59 ± 0.07	7.28 ± 0.02	6.25 ± 0.02	17.00 ± 0.09	16.98 ± 0.04	50.00 ± 0.12	52.36 ± 0.11
THR	12.24 ± 0.01	21.48 ± 0.04	3.39 ± 0.01	3.71 ± 0.01	51.25 ± 0.11	62.42 ± 0.17	0.9 ± 0.01	4.98 ± 0.02	nd	1.25 ± 0.03
TPR	6.23 ± 0.04	6.90 ± 0.02	0.19 ± 0.01	0.25 ± 0.02	40.40 ± 0.10	62.52 ± 0.13	3.91 ± 0.03	4.58 ± 0.03	12.61 ± 0.08	11.56 ± 0.07
VAL	29.20 ± 0.05	61.25 ± 0.21	6.02 ± 0.01	6.67 ± 0.03	90.25 ± 0.21	98.32 ± 0.25	1.80 ± 0.01	22.62 ± 0.08	7.20 ± 0.02	16.25 ± 0.05
Total EAA	244.02 ± 0.91	357.94 ± 0.91	115.01 ± 0.26	132.7 ± 0.45	260.93 ± 0.68	317.31 ± 0.77	205.71 ± 0.50	254.73 ± 0.62	381.01 ± 0.86	443.29 ± 0.91
Non-essential amino acids										
ARG	136.13 ± 0.32	131.18 ± 0.21	65.68 ± 0.09	65.98 ± 0.05	200.01 ± 0.27	215.32 ± 0.22	23.22 ± 0.07	26.58 ± 0.08	35.4 ± 0.07	36.86 ± 0.11
GLN	96.35 ± 0.20	78.65 ± 0.11	80.69 ± 0.11	88.50 ± 0.17	115.93 ± 0.14	105.49 ± 0.11	61.45 ± 0.10	72.56 ± 0.12	61.52 ± 0.08	62.58 ± 0.09
SER	86.51 ± 0.17	86.57 ± 0.13	75.90 ± 0.19	82.90 ± 0.14	143.70 ± 0.19	121.35 ± 0.10	75.54 ± 0.12	85.68 ± 0.13	95.35 ± 0.14	103.5 ± 0.15
ASN	159.20 ± 0.34	136.48 ± 0.28	44.26 ± 0.13	52.90 ± 0.10	369.26 ± 0.23	311.36 ± 0.24	185.52 ± 0.18	195.12 ± 0.19	121.35 ± 0.28	143.5 ± 0.25
1-MHIS	0.90 ± 0.01	11.36 ± 0.03	4.94 ± 0.02	5.06 ± 0.02	14.90 ± 0.09	15.25 ± 0.08	5.44 ± 0.03	6.52 ± 0.03	9.41 ± 0.04	10.26 ± 0.05
HYP	0.12 ± 0.01	0.98 ± 0.01	17.12 ± 0.05	17.24 ± 0.04	98.35 ± 0.10	98.65 ± 0.13	nd	2.36 ± 0.02	2.10 ± 0.01	2.40 ± 0.01
GLY	24.30 ± 0.03	26.58 ± 0.05	43.62 ± 0.09	51.33 ± 0.09	25.94 ± 0.04	26.32 ± 0.06	38.30 ± 0.08	25.65 ± 0.09	71.12 ± 0.10	82.65 ± 0.14
GPR	36.00 ± 0.07	37.42 ± 0.08	0.11 ± 0.01	0.04 ± 0.01	0.68 ± 0.01	2.65 ± 0.01	92.31 ± 0.19	89.56 ± 0.17	124.90 ± 0.22	124.86 ± 0.24
ALA	84.70 ± 0.11	88.14 ± 0.13	115.51 ± 0.21	122.80 ± 0.12	278.32 ± 0.11	289.65 ± 0.27	139.50 ± 0.16	145.69 ± 0.28	90.85 ± 0.13	85.62 ± 0.14
HLY	39.00 ± 0.05	4.56 ± 0.02	nd	nd	nd	4.98 ± 0.02	17.90 ± 0.10	23.71 ± 0.06	82.79 ± 0.16	79.45 ± 0.19
GABA	52.80 ± 0.09	50.31 ± 0.10	165.72 ± 0.11	169.86 ± 0.13	284.93 ± 0.20	287.95 ± 0.19	73.50 ± 0.11	89.65 ± 0.10	95.52 ± 0.11	99.65 ± 0.13
SAR	63.10 ± 0.12	55.71 ± 0.11	0.61 ± 0.01	0.99 ± 0.01	3.36 ± 0.02	15.26 ± 0.06	24.11 ± 0.04	45.68 ± 0.11	203.37 ± 0.21	258.45 ± 0.17
βAIBA	157.00 ± 0.27	162.45 ± 0.19	nd	nd	nd	0.25 ± 0.01	204.5 ± 0.13	230.12 ± 0.21	202.42 ± 0.24	151.23 ± 0.15
ABA	34.71 ± 0.08	35.68 ± 0.11	1.02 ± 0.01	0.86 ± 0.01	2.95 ± 0.02	35.69 ± 0.07	14.51 ± 0.04	26.69 ± 0.07	32.70 ± 0.10	39.12 ± 0.11
ORN	21.53 ± 0.03	25.90 ± 0.07	1.25 ± 0.02	3.25 ± 0.02	4.85 ± 0.03	4.99 ± 0.02	10.60 ± 0.02	12.98 ± 0.08	10.80 ± 0.07	11.08 ± 0.08
PRO	827.75 ± 0.39	962.54 ± 0.32	785.98 ± 0.35	807.08 ± 0.39	1406.14 ± 0.41	1562.35 ± 0.49	852.35 ± 0.45	913.56 ± 0.31	956.99 ± 0.29	985.24 ± 0.33
ASP	53.11 ± 0.11	55.21 ± 0.09	42.81 ± 0.10	52.01 ± 0.09	167.01 ± 0.17	184.53 ± 0.23	20.70 ± 0.09	22.36 ± 0.06	40.55 ± 0.11	42.68 ± 0.11
TYR	24.00 ± 0.10	28.64 ± 0.01	6.55 ± 0.03	7.01 ± 0.02	6.52 ± 0.01	11.65 ± 0.05	3.00 ± 0.02	4.12 ± 0.02	36.50 ± 0.08	34.56 ± 0.11
GLU	24.62 ± 0.07	28.65 ± 0.09	29.15 ± 0.03	31.61 ± 0.08	9.82 ± 0.05	8.65 ± 0.04	24.36 ± 0.09	28.65 ± 0.10	270.20 ± 0.14	281.35 ± 0.16
TRP	5.17 ± 0.02	1.29 ± 0.02	2.25 ± 0.02	4.56 ± 0.03	12.61 ± 0.09	10.19 ± 0.06	12.00 ± 0.07	5.61 ± 0.02	0.90 ± 0.01	1.22 ± 0.01
Total NEAA	1927.00 ± 2.59	2008.30 ± 2.16	1483.17 ± 1.58	1563.98 ± 1.52	3145.28 ± 2.18	3312.53 ± 2.46	1878.81 ± 2.09	2052.85 ± 2.25	2544.74 ± 2.59	2636.26 ± 2.73
Total AA	2170.9 ± 3.50	2366.24 ± 3.07	1598.19 ± 1.81	1696.68 ± 1.97	3406.2± 2.86	3629.84 ± 3.23	2084.42 ± 2.59	2307.57 ± 2.87	2925.41 ± 3.45	3079.53 ± 3.64

BP = bee-collected pollen, FBP = fermented bee-collected pollen. Results represent the mean ± standard deviation of three independent determinations; nd = not detected, EAA = essential amino acid, NEAA = non-essential amino acid, AA = amino acid, HIS = histidine, ISO = isoleucine, LEU = leucine, LYS = lysine, MET = methionine, PHE = phenylalanine, THR = threonine, TRP = tryptophan, VAL = valine, ARG = arginine, GLN = glutamine, SER = serine, ASN = aspargin, 1-MHIS = 1-methyl-histidine, HYP = 4-hydroxyproline, GLY = glycine, GPR = glycine–proline (dipeptide), ALA = alanine, HLY = hydroxylisine, GABA = gamma-aminobutyric acid, SAR = sarcosine, βAIBA = beta-aminoisobutyric acid, ABA = alpha-aminobutyric acid, ORN = ornithine, PRO = proline, ASP = aspartic acid, TYR = tyrosine, GLU = glutamic acid, TRP = thiaprolin.

**Table 6:** Quantification of the phenolic compounds (µg/g of extract) present in BP and FBP.

Identified Compound	Sample, mg/mL (dw)									
	BP1	FBP1	BP2	FBP2	BP3	FBP3	BP4	FBP4	BP5	FBP5
Caffeic acid	13.70 ± 0.04 ^b^	17.67 ± 0.09 ^c^	11.09 ± 0.05 ^d^	14.63 ± 0.07 ^e^	0.11 ± 0.01 ^f^	6.09 ± 0.08 ^g^	11.56 ± 0.02 ^d^	16.68 ± 0.04 ^h^	16.50 ± 0.03 ^h^	19.97 ± 0.06 ^a^
Chlorogenic acid	5.20 ± 0.11 ^e^	0.22 ± 0.02 ^c^	3.46 ± 0.06 ^f^	0.15 ± 0.02 ^c^	8.20 ± 0.103 ^a^	0.94 ± 0.07 ^b^	0.70 ± 0.03 ^b^	0.21 ± 0.05 ^c^	0.92 ± 0.04 ^b^	0.55 ± 0.05 ^d^
*trans*-*p*-Coumaric acid	17.67 ± 0.13 ^h^	59.40 ± 0.19 ^i^	26.85 ± 0.22 ^j^	62.97 ± 0.12 ^a^	11.21 ± 0.02 ^b^	42.28 ± 0.25 ^c^	13.35 ± 0.04 ^d^	25.02 ± 0.11 ^e^	0.43 ± 0.02 ^f^	45.43 ± 0.09 ^g^
Ellagic acid	24.76 ± 0.17 ^d^	37.34 ± 0.12 ^e^	nd	nd	nd	nd	154.90 ± 0.2 ^g^	266.40 ± 0.22 ^a^	74.36 ± 0.17 ^b^	107.62 ± 0.21 ^c^
Ferulic acid	7.23 ± 0.08 ^d^	14.07 ± 0.10 ^e^	14.41 ± 0.17 ^e^	19.92 ± 0.11 ^f^	26.38 ± 0.09 ^g^	36.43 ± 0.18 ^a^	nd	nd	2.67 ± 0.02 ^b^	5.43 ± 0.08 ^c^
Rosmarinic acid	0.22 ± 0.03 ^a^	0.07 ± 0.02 ^a^	0.08 ± 0.02 ^a^	nd	0.22 ± 0.02 ^a^	0.23 ± 0.06 ^a^	nd	nd	nd	nd
Salicylic acid	nd	nd	nd	nd	127.75 ± 0.05 ^e^	132.55 ± 0.07 ^a^	29.85 ± 0.02 ^b^	32.51 ± 0.08 ^c^	0.26 ± 0.07 ^d^	0.93 ± 0.10 ^d^
Apigenin	1.01 ± 0.02 ^d^	14.05 ± 0.09 ^e^	2.44 ± 0.07 ^f^	9.53 ± 0.08 ^g^	1.39 ± 0.04 ^d^	12.29 ± 0.03 ^h^	6.81 ± 0.06 ^i^	17.36 ± 0.04 ^a^	1.49 ± 0.02 ^b^	5.37 ± 0.09 ^c^
Chrysin	1.35 ± 0.04 ^b^	1.14 ± 0.03 ^b^	0.66 ± 0.05 ^c^	0.69 ± 0.02 ^c^	2.02 ± 0.02 ^d^	4.46 ± 0.03 ^e^	0.30 ± 0.01 ^c^	0.58 ± 0.01 ^c^	3.86 ± 0.05 ^e^	9.91 ± 0.03 ^a^
Salicin	0.03 ± 0.01 ^b^	0.13 ± 0.03 ^b^	0.45 ± 0.06 ^c^	0.95 ± 0.06 ^c^	0.05 ± 0.01 ^b^	1.67 ± 0.02 ^d^	0.43 ± 0.03 ^c^	3.99 ± 0.07 ^a^	0.06 ± 0.01 ^b^	0.36 ± 0.01 ^c^
Quercetin-3-*O*-galactoside	21.52 ±0.05 ^e^	22.88 ± 0.09 ^e,h^	13.79 ± 0.11 ^f^	17.37 ± 0.11 ^g^	23.16 ± 0.05 ^h^	36.97 ± 0.07 ^a^	nd	4.15 ± 0.01 ^b^	5.96 ± 0.10 ^c^	10.89 ± 004 ^d^
Kaempferol	72.52 ± 0.26 ^b^	85.68 ± 0.37 ^c^	0.26 ± 0.07 ^d^	22.77 ± 0.06 ^e^	112.21 ± 0.22 ^f^	192.48 ± 0.26 ^g^	6.22 ± 0.02 ^h^	65.34 ± 0.24 ^i^	234.78 ± 0.14 ^j^	336.55 ± 0.31 ^a^
Luteolin	12.81 ± 0.11 ^g^	82.39 ± 0.22 ^a^	12.76 ± 0.11 ^b^	18.94 ± 0.11 ^c^	nd	6.90 ± 0.03 ^d^	nd	nd	0.13 ± 0.04 ^e^	3.73 ± 0.05 ^f^
Luteolin-7-O-glucosid	0.08 ± 0.01 ^a^	0.14 ± 0.05 ^a^	0.13 ± 0.04 ^a^	0.05 ± 0.01 ^a^	0.04 ± 0.01 ^a^	0.03 ± 0.01 ^a^	nd	0.03 ± 0.01 ^a^	nd	nd
Myricetin	94.73 ± 0.18 ^b^	104.00 ± 0.21 ^a^	nd	nd	nd	nd	nd	nd	nd	nd
Naringenin	0.03 ± 0.01 ^b^	0.87 ± 0.06 ^b^	0.55 ± 0.02 ^b^	4.94 ± 0.03 ^c^	nd	nd	0.73 ± 0.02 ^b^	9.77 ± 0.02 ^a^	0.18 ± 0.03 ^b^	5.31 ± 0.02 ^c^
Quercetin	115.36 ± 0.21 ^i^	324.63 ± 0.33 ^a^	104.62 ± 0.32 ^b^	306.02 ± 0.29 ^c^	nd	57.59 ± 0.01 ^d^	31.31 ± 0.03 ^e^	75.05 ± 0.11 ^f^	8.70 ± 0.09 ^g^	39.22 ± 0.08 ^h^
Quercetin-3-*O*-rutinoside	33.54 ± 0.02 ^b^	125.94 ± 0.12 ^c^	71.75 ± 0.25 ^d^	139.24 ± 0.22 ^e^	34.97 ± 0.07 ^b^	138.53 ± 0.13 ^e^	134.23 ± 0.34 ^f^	187.81 ± 0.17 ^g^	94.67 ± 0.11 ^h^	167.24 ± 0.14 ^a^
Vitexin	0.46 ± 0.02 ^b^	0.62 ± 0.05 ^b^	nd	nd	6.73 ± 0.05 ^c^	15.13 ± 0.11 ^d^	nd	nd	1.23 ± 0.03 ^e^	34.78 ± 0.13 ^a^

BP = bee-collected pollen, FBP = fermented bee-collected pollen, nd = not detected. dw = dry weight. Values are represented as means ± standard deviations. Within the same row, different letters indicate significant differences (*p* < 0.05). The results are in accordance with those obtained for polyphenols and total flavonoids for these samples, showing that the fermentation positively influenced the amounts of phenolic compounds. It is well known that phenolic compounds have antibacterial and antioxidant qualities. Identifying and quantifying these compounds helps assess the health benefits of pollen; moreover, the analysis of phenolic compounds helps to identify the changes occurring during lactic fermentation.

**Table 7:** Inhibition zones of BP and FBP samples against different microorganisms.

Bacterial Strain	Inhibition Diameters (mm)									
	BP1	FBP1	BP2	FBP2	BP3	FBP3	BP4	FBP4	BP5	FBP5
Gram-Positive Bacteria										
*S. aureus*	12.78 ± 0.65 ^b^	21.32 ± 0.35 ^c^	13.33 ± 0.47 ^b^	22.25 ± 0.36 ^d^	15.21 ± 0.24 ^e^	24.56 ± 0.25 ^a^	18.32 ± 0.50 ^f^	24.50 ± 0.25 ^a^	20.50 ± 0.24 ^c^	25.15 ± 0.50 ^a^
*E. faecalis*	20.90 ± 0.11 ^b^	25.25 ± 0.38 ^c^	16.47 ± 0.28 ^d^	20.28 ± 0.31 ^b,e^	18.31 ± 0.33 ^f^	22.61 ± 0.45 ^g^	19.57 ± 0.38 ^e^	26.39 ± 0.50 ^g^	20.43 ± 0.35 ^b^	29.58 ± 0.25 ^a^
Gram-Negative Bacteria										
*E. coli*	15.33 ± 0.83 ^b^	20.25 ± 0.25 ^c^	10.50 ± 0.55 ^d^	13.33 ± 0.54 ^e^	11.47 ± 0.62 ^d,f^	12.59 ± 0.57 ^e,f^	15.43 ± 0.23 ^b^	20.28 ± 0.44 ^c^	16.21 ± 0.78 ^b^	27.11 ± 0.73 ^a^
*P. aeruginosa*	4.25 ± 0.11 ^b^	9.50 ± 0.34 ^c^	4.37 ± 0.28 ^b^	10.28 ± 0.50 ^c^	5.55 ± 0.57 ^d^	9.67 ± 0.31 ^c^	6.72 ± 0.46 ^d^	11.68 ± 0.67 ^e^	8.50 ± 0.23 ^c^	13.21 ± 0.26 ^a^
Yeast										
*C. albicans*	7.33 ± 0.42 ^b^	9.25 ± 0.75 ^c^	6.25 ± 0.55 ^b,d^	9.16 ± 0.34 ^c^	5.25 ± 0.14 ^d^	13.57 ± 0.27 ^e^	9.36 ± 0.16 ^c^	14.45 ± 0.35 ^e^	10.65 ± 0.23 ^f^	15.57 ± 0.17 ^a^

Values are represented as means ± standard deviations. Within the same row, different letters indicate significant differences (*p* < 0.05).

**Table 8:** Minimum inhibitory concentrations (MICs) of BP and FBP samples against different microorganisms.

Bacterial Strain	Minimum Inhibitory Concentration (MIC) (mg/mL)									
	BP1	FBP1	BP2	FBP2	BP3	FBP3	BP4	FBP4	BP5	FBP5
Gram-positive bacteria										
*S. aureus*	3.12	1.56	3.12	1.56	1.56	0.78	0.78	0.39	0.78	0.39
*E. faecalis*	6.25	3.12	6.25	3.12	3.12	1.56	1.56	0.78	0.78	0.39
Gram-negative bacteria										
*E. coli*	6.25	3.12	3.12	1.56	3.12	1.56	3.12	0.78	3.12	0.39
*P. aeruginosa*	25.00	12.50	25.00	12.50	25.00	12.50	12.50	6.25	12.50	6.25
Yeast										
*C. albicans*	6.25	3.12	12.50	6.25	6.25	1.56	3.12	1.56	1.56	0.78

## Data Availability

The data used to support the findings of this study are included within the article.
